# Radiofrequency ablation reduces esophageal hypersensitivity in refractory non-erosive reflux disease

**DOI:** 10.3389/fmed.2026.1687564

**Published:** 2026-02-13

**Authors:** Yan Chen, Xin Lu, Ming Cheng, Mingrui Cui, Xuejun Wen, Kai Mu, Ping Wang, Ying-Jian Zhang

**Affiliations:** 1 Department of Gastroenterology, The First Affiliated Hospital, College of Clinical Medicine, Hospital of Science and Technology, Luoyang, Henan, China; 2 Henan Medical Key Laboratory of Gastrointestinal Microecology and Hepatology, Henan University of Science and Technology, Luoyang, Henan, China; 3 International Institute for Biomedical Biomaterials (I2BM), Zhengzhou, China; 4 Department of Chemical and Life Science Engineering, School of Engineering, Virginia Commonwealth University, Richmond, VA, United States; 5 The First Affiliated Hospital, College of Clinical Medicine, Henan University of Science and Technology, Luoyang, Henan, China; 6 Department of Public Health, School of Basic Medical Sciences, Henan University of Science and Technology, Luoyang, Henan, China

**Keywords:** esophageal sensitivity, heartburn, PAR2, refractory non-erosive gastroesophageal reflux disease, TRPV1

## Abstract

Refractory non-erosive gastroesophageal reflux disease (rNERD) involves complex interactions between esophageal sensitivity receptors (PAR2/TRPV1) and persistent proton pump inhibitor (PPI)-refractory symptoms. This study exclusively employed radiofrequency ablation (RFA) as the uniform endoscopic intervention. All 20 enrolled rNERD patients underwent standardized RFA procedures; no participants underwent mucosal resection or ligation procedures. Post-intervention analysis demonstrated significant reductions in PAR2/TRPV1 expression (PAR2: 81.8% → 35.5%; TRPV1: 1.6 → 0.9 by Western blot) and inflammatory infiltration. Symptom improvement correlated with receptor downregulation (Reflux Symptom Index (RSI): 14.6 → 5.0; Gastroesophageal Reflux Disease Questionnaire (GERD-Q): 14.0 → 7.6). These findings indicate that endoscopic RFA alleviates rNERD symptoms through dual mechanisms: anatomical correction and esophageal hypersensitivity modulation via PAR2/TRPV1 pathways.

## Introduction

Gastroesophageal reflux disease (GERD) represents one of the most prevalent upper gastrointestinal disorders, characterized primarily by reflux of gastric contents leading to acid regurgitation, heartburn, and extraesophageal manifestations such as chronic cough and asthma-like symptoms (1, 2). Proton pump inhibitors (PPIs) serve as first-line therapy for GERD and effectively relieve typical symptoms, achieving high cure rates for reflux esophagitis (3–5). However, with widespread PPI use, clinicians observe hat a subset of GERD patients shows incomplete or absent symptom relief despite standard once-daily PPI dosing—a condition termed refractory GERD (rGERD) or PPI treatment failure (6, 7). Refractory GERD (rGERD) is defined as persistent symptoms despite standard PPI therapy (8). “Standard PPI therapy” refers to at least 8 weeks of once-daily administration at recommended doses. The refractoriness is distinguished from inadequate treatment duration (<8 weeks), medication non-adherence, or inappropriate initial diagnosis. Endoscopic findings serve primarily for classifying GERD subtypes (erosive vs. non-erosive) but do not solely determine treatment refractoriness, which requires comprehensive assessment including functional testing. These refractory cases substantially compromise patients’ quality of life through persistent symptomatology and recurrent healthcare utilization. The pathogenesis of rGERD involves multiple factors, including esophageal dysmotility, impaired mucosal integrity, psychological comorbidities, and particularly esophageal hypersensitivity (9–11). Endoscopically, most rGERD patients show no mucosal damage (non-erosive reflux disease), with only a minority exhibiting reflux esophagitis (12). While esophageal dysmotility is common in rGERD (13), its specific patterns require further investigation.

Baseline impedance measurement has emerged as an important marker of esophageal mucosal integrity in GERD (14). Studies demonstrate that baseline impedance levels correlate with abnormalities in esophageal acid exposure, showing diagnostic value superior to PPI therapeutic response alone (15). After studying the relationship between esophageal impedance baseline and epithelial cell gap in (Non-erosive reflux disease) NERD patients, it is concluded that there is a negative correlation between the former and the latter (16). Furthermore, histological analysis revealed an inverse correlation between dilated intercellular spaces and baseline impedance, establishing this parameter as a reliable marker of mucosal integrity (15). Therefore, baseline impedance may be an important diagnostic tool, which is helpful to distinguish whether GERD or heartburn in patients with PPI refractory reflux is only functional.

As clinically established, esophageal hypersensitivity represents one key mechanism underlying heartburn symptoms. This condition is primarily induced by abnormal acid exposure in the esophagus, which subsequently influences baseline impedance levels and contributes to mucosal damage. Therefore, esophageal baseline impedance measurement serves as an appropriate method for evaluating acid sensitivity in patients presenting with heartburn (17, 18). In order to further study the mechanism of heartburn in GERD, some researchers have investigated immunohistochemical markers, including a study of 94 GERD patients, which showed that the expression of PAR-2 was significantly related to basal cell proliferation, dilated intercellular spaces, and inflammatory cell count, which was also related to heartburn (19). Protease-activated receptor-2 (PAR-2) has been shown to be highly expressed in both erosive and non-erosive GERD, where it may mediate visceral hypersensitivity (19). Esophageal visceral hypersensitivity also plays an important role in the mechanism of heartburn symptoms. By monitoring reflux events, clinical studies have found that the number and degree of acid exposure abnormalities in patients with NERD are less than those in patients with erosive esophagitis, but the level of heartburn symptoms in both patients is the same, which is closely related to NERD and visceral hypersensitivity (VH) (20).

While the pathogenesis of GERD remains incompletely understood, resulting in suboptimal therapeutic outcomes (21, 22). Therefore, more clearly and systematically explaining the pathogenesis of GERD, exploring potential effective therapeutic targets and choosing the best treatment plan have gradually become the focus of scholars’ attention (23). Current domestic and international research on esophageal hypersensitivity and endoscopic interventions for refractory gastroesophageal reflux disease (rGERD) has mainly concentrated on two aspects: the distribution patterns of esophageal sensitivity markers among various GERD clinical subtypes and anatomical improvements of esophageal architecture following therapeutic procedures. However, this body of work has largely overlooked the critical relationship between endoscopic interventions and alterations in esophageal sensitivity. This study employed a comprehensive multimodal approach combining high-resolution esophageal manometry (HRM) and 24-h pH-impedance monitoring to evaluate patients with refractory non-erosive reflux disease (rNERD). Our findings demonstrate that esophageal hypersensitivity likely plays a pivotal role in disease pathogenesis. Using endoscopic interventions, HE staining, immunohistochemistry, western blot, ELISA, (Reflux Symptom Index)RSI and GERD-Q questionnaires, we systematically evaluated alterations in esophageal sensitivity following GERD treatment. The results confirm that esophageal hypersensitivity constitutes a key pathophysiological mechanism in rNERD, providing objective evidence for clinical diagnosis and therapeutic evaluation while identifying potential targets for future GERD treatment strategies. This study employed a retrospective observational design to investigate the relationship between endoscopic therapy and alterations in esophageal sensitivity in patients with refractory non-erosive gastroesophageal reflux disease. It is important to note that this investigation focused specifically on radiofrequency ablation outcomes. The exclusion of other endoscopic modalities (mucosal resection, ligation) was intentional to maintain intervention consistency and facilitate clear interpretation of therapeutic effects.

Refractory non-erosive gastroesophageal reflux disease (rNERD) involves complex interactions between esophageal sensitivity receptors (PAR2/TRPV1) and persistent proton pump inhibitor (PPI)-refractory symptoms. To address this, the present study aimed to evaluate the effects of endoscopic radiofrequency ablation (RFA) on esophageal hypersensitivity markers (PAR2 and TRPV1) in patients with rNERD and to assess the correlation between changes in these markers and clinical symptom improvement. We hypothesized that RFA would downregulate the expression of PAR2 and TRPV1, leading to a reduction in esophageal hypersensitivity and symptomatic relief. This study exclusively employed RFA as the uniform endoscopic intervention due to its documented safety profile and potential to modify esophageal tissue properties, which may influence hypersensitivity.”

## Materials and methods

### Study design

This was a retrospective observational study investigating the effect of endoscopic radiofrequency ablation (RFA) on esophageal hypersensitivity in refractory non-erosive reflux disease (rNERD). To ensure a homogeneous intervention for clear mechanistic interpretation, we exclusively included patients who underwent RFA, excluding those receiving other endoscopic therapies (e.g., mucosal resection, ligation).

From the rGERD patient pool defined above, the specific diagnosis of refractory NERD for intervention required fulfillment of all the following additional stringent criteria: (1) Persistent typical and/or atypical reflux symptoms (assessed via Reflux Symptom Index [RSI] ≥ 13 and Gastroesophageal Reflux Disease Questionnaire [GERD-Q] ≥ 8) despite a documented ≥ 8-week course of standard-dose once-daily PPI therapy; (2) Endoscopic confirmation of non-erosive mucosa (Los Angeles classification grade 0); (3) Objective evidence of reflux on 24-h pH-impedance monitoring (acid exposure time >6% and/or total number of reflux episodes >80). Patients meeting these criteria were offered and subsequently underwent RFA.”

### Definition of Gastroscophageal reflux disease (GERD)

In this study, the diagnosis of GERD was established based on the widely recognized Montreal definition and classification from the global evidence-based consensus (2). Specifically, patients were required to meet at least one of the following criteria:

1. Presence of troublesome reflux-related symptoms that impacted quality of life, including typical symptoms (heartburn and/or regurgitation) and/or atypical/extra-esophageal symptoms (such as chronic cough, sensation of a lump in the throat, or asthma-like symptoms).
2. Endoscopic evidence of reflux esophagitis according to the Los Angeles (LA) classification system (grades A–D).
3. Objective pathological reflux documented by 24-h pH-impedance monitoring, defined as either an acid exposure time (AET) > 6% and/or a positive symptom-reflux association (symptom association probability, SAP ≥ 95%).

### Definition of refractory gastroesophageal reflux disease (rGERD)

Refractory GERD (rGERD) was operationally defined as: Patients fulfilling the above GERD criteria who reported persistent reflux symptoms despite an adequate trial of proton pump inhibitor (PPI) therapy. For this study, “adequate PPI therapy” was strictly defined as: a minimum of 8 weeks of continuous, once-daily administration of a standard-dose PPI (e.g., omeprazole 20 mg or equivalent). To ensure adherence and exclude inadequate treatment duration, PPI use was meticulously recorded and verified from patient histories.

“Symptom persistence” was determined through a combination of:

1. Subjective assessment: Scores above the validated thresholds on standardized questionnaires (Reflux Disease Questionnaire (RDQ) and/or the Reflux Symptom Index (RSI) and GERD-Q as described in the refractory NERD criteria).
2. Objective confirmation: Where available, persistent abnormal findings on 24-h pH-impedance monitoring (e.g., pathological acid/non-acid reflux) conducted off PPI therapy (after a 2-week washout period as per our protocol) provided supporting evidence for refractoriness, particularly for distinguishing from functional heartburn.

### Patient grouping strategy

Based on these definitions, the enrolled patient pool (*n* = 74) was stratified as follows:

#### GERD group

Patients meeting the GERD criteria whose symptoms were adequately controlled (RDQ scores below threshold and/or patient-reported significant improvement) after the documented course of standard PPI therapy. This group was further subdivided endoscopically into Non-Erosive Reflux Disease (NERD) and Reflux Esophagitis (RE) subgroups.

#### rGERD group

Patients meeting the GERD criteria who continued to report persistent symptoms (as defined above) despite the adequate PPI trial. This group was also subdivided into Refractory NERD and Refractory RE based on endoscopic findings (LA grade 0 vs. LA grade A-D).

The core intervention cohort for the pre-post RFA analysis consisted of 20 patients from the Refractory NERD subgroup who consented to and underwent the endoscopic radiofrequency ablation procedure.

### Clinical data collection

We retrospectively analyzed clinical data from patients aged 18–80 years who presented to the Department of Gastroenterology at the New District Hospital of Henan University of Science and Technology between September 2017 and September 2018. Eligible participants presented with symptoms such as acid regurgitation, heartburn, and eructation, or esophageal foreign body sensation and completed standardized assessments: a general status questionnaire, Reflux Disease Questionnaire (RDQ), extraesophageal symptom survey, and PPI usage record. Patients were divided into GERD and refractory GERD (rGERD) groups based on symptom persistence despite standard PPI therapy. The GERD group comprised both non-erosive reflux disease (NERD) and reflux esophagitis (RE) cases, while the rGERD group included both refractory NERD and refractory RE subgroups. All participants underwent HRM and 24-hour pH-impedance monitoring. According to the symptom scale and PPI application, combined with relevant diagnostic criteria, 74 cases were collected, including 32 males and 42 females. Inclusion criteria: (1) Age 18–80, regardless of sex; (2) Patients with typical symptoms such as acid reflux and heartburn, with or without epigastric symptoms and atypical symptoms such as belching, abdominal distension, throat discomfort and cough; (3) PPI drugs should be stopped for 2 weeks and prokinetic drugs for 1 week before HRM and 24 h pH- hour pH-impedance joint monitoring. Exclusion criteria: (1) upper gastrointestinal endoscopy, HRM and 24 h pH- hour pH-impedance monitoring were not performed; (2) Previous history of esophageal and gastric surgery; (3) Complicated with digestive tract ulcer, malignant tumor, heart and lung diseases and severe liver and kidney diseases; (4) Pregnant and lactating women; (5) Those who abuse alcohol or drugs and cannot state their illness; (6) achalasia of cardia, diabetes and other diseases that affect gastrointestinal motility; (7) Patient data is incomplete, and there is no specific information about PPI use. This preliminary investigation employed a consecutive sample of 20 patients undergoing endoscopic RFA specifically for refractory non-erosive reflux disease (rNERD). While this sample size provides initial mechanistic insights, we acknowledge it limits statistical power for subgroup analyses. Post-hoc power analysis confirmed 80% power to detect large effect sizes (Cohen’s *d* > 0.8) for primary outcomes. The diagnosis of refractory status was confirmed through multimodal evaluation, integrating: (1) persistent symptoms following complete PPI course; (2) objective functional parameters (HRM, 24-h pH-impedance); and (3) symptomatic assessment using validated instruments (RSI, GERD-Q). Endoscopy provided non-erosive confirmation in rNERD cases. This preliminary, hypothesis-generating study enrolled a consecutive sample of 20 patients with rNERD undergoing RFA. A post-hoc power analysis indicated that with *n* = 20 and *α* = 0.05, the study had 80% power to detect large effect sizes (Cohen’s *d* > 0.8) for pairwise comparisons of primary molecular outcomes (e.g., PAR2, TRPV1 expression changes).

### Preoperative specimen collection

Twenty patients with the above-mentioned refractory and non-erosive gastroesophageal reflux disease who are going to undergo endoscopic treatment (radiofrequency ablation) were scored with RSI and GERD-Q at the time of admission. All 20 enrolled patients with refractory non-erosive gastroesophageal reflux disease received radiofrequency ablation treatment, with no patients undergoing mucosal resection or ligation procedures. This uniform intervention approach ensured treatment homogeneity across the study cohort. Serum samples were collected and stored at −80 °C, while two tissue specimens (obtained 2 cm above the esophagogastric junction or lesion site) were preserved: one in formaldehyde at room temperature for histopathological analysis, and another snap-frozen in cryotubes at −80 °C for molecular studies. All specimens were obtained following provision of informed consent and approved by the Ethics Committee of Henan University of Science and Technology.

### Postoperative specimen collection

Six months after endoscopic treatment, patients with GERD were followed up by telephone and subsequently underwent clinical reassessment. During follow-up visits, Reflux Symptom Index (RSI) and GERD-Q questionnaires were administered to evaluate treatment outcomes. Serum samples were collected and stored at −80 °C for subsequent analysis. Additionally, two tissue specimens (each obtained from either the esophageal lesion site or 2 cm above the esophagogastric junction) were preserved using dual methods: one sample was fixed in formaldehyde for room temperature storage, while the other was immediately snap-frozen in cryotubes and maintained at −80 °C for future molecular studies. This standardized protocol ensured consistent sample integrity across all study participants.

### Clinical symptom related score

All 74 patients enrolled in the study were assessed using the GERD-Q questionnaire. Additionally, among these participants, 20 diagnosed with refractory non-erosive gastroesophageal reflux disease (rNERD) underwent both preoperative and postoperative evaluation through standardized symptom assessment tools, including the Reflux Symptom Index (RSI) and GERD-Q scoring systems.

### High-resolution esophageal manometry (HRM)

Fill and connect the water tank to the catheter. Enter patient info into software, select esophageal manometry mode with a 24-channel catheter, and start pressure measurement. Zero and flush the system at high pressure (80 mmHg) to remove air bubbles. Calibrate at low and high pressures, position catheter at gastric baseline (supine), and zero again before insertion. With the patient seated, insert lubricated catheter through nostril to 2 cm below LES and secure. Have patient lie down, start pressure measurement: during initialization, patient performs deep breathing for diaphragmatic localization. Begin formal measurements after ensuring patient adaptation and stabilization of UES and LES pressure profiles, then input catheter distance. Record 30-s baseline resting pressure, label appropriately. Conduct sequential swallow tests: ten 5 mL water swallows (single swallows only; repeat if compromised), five 10 mL water swallows, and five rapid 2 mL water boluses within one second, all annotated. After recordings, carefully remove catheter before stopping system to preserve data.

### 24-h pH-impedance monitoring

The equipment was prepared with a new AA battery, electrode catheter, pH 4.0 and pH 7.01 calibration solutions, data recorder, carrying pouch. Install battery in recorder, place in pouch, and connect catheter to recorder’s color-coded interface. Start calibration mode, set date/time, and soak catheter for 60 s. Dry catheter, immerse in pH 4.0 buffer for ~3 min, rinse, and dry. Repeat with pH 7.01 buffer. Lubricate distal 20 cm of catheter. With patient seated, gently insert catheter through nasal cavity to ~15 cm into pharynx, instruct patient to swallow to advance catheter into esophagus. Position catheter 5 cm above LES (locate by advancing into stomach, withdrawing while monitoring pH changes, cross-referencing with manometry), fix catheter, adjust strap, and press “Start Recording.” Record start time, instruct patient to fill in record form. After 24 h, remove device, connect recorder to computer, import data into analysis software, verify patient info, save, and confirm data integrity. Observe patient for 30 min, transfer data to patient file, open waveform interface, and allow system to auto-analyze and generate report.

### HE staining

Collected esophageal tissues were processed into paraffin-embedded sections. The sections underwent a standard deparaffinization and rehydration procedure using xylene and graded ethanol solutions. After rinsing with tap water, they were stained with hematoxylin for 3–5 min, followed by eosin counterstaining for 5 min. The sections were then dehydrated and sealed with mounting medium for microscopic imaging and histological analysis.

### Immunohistochemistry

Paraffin-embedded sections were deparaffinized and subjected to antigen retrieval in a citrate-based buffer (pH 6.0) using a retrieval chamber. Endogenous peroxidase activity was blocked with 3% hydrogen peroxide solution. The sections were blocked with 3% bovine serum albumin (BSA) at room temperature for 30 min. After removing the blocking solution, Sections were incubated overnight at 4 °C with rabbit anti-human PAR2 polyclonal antibody (Abcam, ab180953, 1:200 dilution) or rabbit anti-human TRPV1 monoclonal antibody (Cell Signaling Technology, #8240, 1:100 dilution), and the sections were incubated overnight at 4 °C in a humidified chamber. Following incubation, the slides were washed three times (5 min each) in PBS (pH 7.4). After brief drying, they were incubated with an HRP-conjugated secondary antibody (matched to the primary antibody species) at room temperature for 50 min. Chromogenic development was carried out using 3,3′-diaminobenzidine (DAB), followed by hematoxylin counterstaining. The stained sections were dehydrated, mounted, and prepared for microscopic imaging and analysis.

### Western blotting

Tissue samples were homogenized in RIPA lysis buffer (Beyotime, P0013B) containing protease inhibitors on ice and centrifuged at 12,000 rpm, 4 °C for 10 min to collect the supernatant. Protein concentration was determined using a BCA protein assay kit (Beyotime, P0010S). Proteins (30 μg per lane) were separated by 10% SDS-PAGE electrophoresis and transferred to a PVDF membrane at 300 mA for 30 min. The membrane was blocked with 5% non-fat milk in TBST for 1 h at room temperature, then incubated with primary antibodies against PAR2 (Abcam, ab180953, 1:1000 dilution) or TRPV1 (Cell Signaling Technology, #8240, 1:1000 dilution) on a shaking platform at 4 °C overnight‌. After washing with TBST (3 × 10 min), the membrane was incubated with HRP-conjugated goat anti-rabbit secondary antibody (Servicebio, GB23303, 1:5000 dilution) for 1 h at room temperature. In the darkroom, ECL reagents A and B (Beyotime, P0018FS) were mixed in equal volumes and applied for chemiluminescent detection. The exposed film was scanned, archived, and the optical density values of target protein bands were quantitatively analyzed using the Alpha imaging software system.

### Enzyme-linked immunosorbent assay (ELSIA)

The assay was performed strictly according to the manufacturer’s instructions of the Human PAR2/Protease Activated Receptor 2 ELISA Kit (CUSABIO, CSB-E17121h) and the Human TRPV1/VR1 ELISA Kit (CUSABIO, CSB-E13515h). Briefly, the capture antibody (provided in the kit) was diluted with carbonate coating buffer to achieve a protein concentration of 1–10 μg/mL. A 100 μL aliquot of this solution was dispensed into each well of the polystyrene plate and incubated overnight at 4 °C. Following plate sealing and subsequent washing steps (using the wash buffer provided), 100 μL of standard or serum sample (diluted 1:2 with sample diluent provided) was added to each well and incubated at 37 °C for 2 h with the plate sealed. After additional washing, 100 μL of diluted biotinylated detection antibody working solution (provided) was introduced and allowed to incubate at 37 °C for 1 h. Subsequent to further washing, 100 μL of diluted HRP-streptavidin conjugate (provided) was added and incubated at 37 °C for 30 min under light-protected conditions. For chromogenic development, 100 μL of TMB substrate solution (provided) was added to each well and incubated at 37 °C in the dark for 15 min. The reaction was terminated by adding 100 μL of stop solution (2 M sulfuric acid, provided), resulting in a color transition from blue to yellow. Within 10 min of stopping the reaction, the optical density (OD) at 450 nm was measured using a microplate reader (BioTek, ELx800), with a reference wavelength of 630 nm. Absorbance values from all test wells were recorded and the concentrations of PAR2 and TRPV1 in serum were calculated based on the standard curve generated for each assay.

### Blinding procedures

All histological and molecular analyses were performed by evaluators blinded to patient group assignment and treatment status. Sample processing and data collection were conducted independently to prevent bias in interpretation.

### Adherence to STROBE guidelines

This study was conducted and reported in accordance with the STROBE (Strengthening the Reporting of Observational studies in Epidemiology) statement for observational cohort studies. The completed STROBE checklist has been included as a supplementary file to ensure comprehensive methodological transparency.

While patient allocation to RFA treatment followed clinical indications rather than randomization, all laboratory assays (histopathology, immunohistochemistry, Western blot, ELISA) were performed by personnel blinded to clinical data and temporal sequence.

### Statistical analysis

All statistical analyses were performed using SPSS software (version 23.0). Continuous variables with normal distribution and homogeneity of variance were expressed as mean ± standard deviation (x̄ ± s) and compared between groups using independent samples t-test. Non-normally distributed continuous data were presented as median with interquartile range (lower quartile, median, upper quartile) and analyzed using nonparametric Mann–Whitney U test. Categorical variables were summarized as frequencies and percentages. A two-tailed *p*-value <0.05 was considered statistically significant for all comparisons. To address potential confounding factors, all analyses were adjusted for age, sex, body mass index, and preoperativesymtom scores using multivariate regression models. To address sample size constraints, we employed effect size calculations (Cohen’s d) alongside traditional *p*-value thresholds, and all models were adjusted for clinical covariates. Multivariate regression models adjusting for age, sex, BMI, and preoperative symptom scores were employed to address potential confounding.

## Results

### Comparison of general data

Based on the Reflux Disease Questionnaire (RDQ) scores, PPI usage patterns, and established diagnostic criteria, the 74 enrolled patients were stratified into GERD and refractory GERD (rGERD) groups. Subsequent endoscopic evaluation further classified the GERD cohort into: 36 non-erosive reflux disease (NERD) cases (48.6%; 14 male, 22 female; age range 37–66 years; mean age 50.69 ± 7.18 years). The rGERD group comprised 28 refractory NERD cases (37.8%; 15 male, 13 female; age range 28–66 years; mean age 49.21 ± 10.43 years) and 10 refractory reflux esophagitis (RE) cases (13.5%; 4 male, 6 female; age range 33–77 years; mean age 55.60 ± 13.70 years). There was no significant difference in sex, age and BMI among the groups (*p* > 0.05). There was no significant difference in RDQ score ratio between refractory NERD patients and NERD patients (*p* = 0.881). There was no significant difference in RDQ scores between refractory NERD group and refractory RE group (*p* = 0.351), as detailed in Table 1.

**Table 1:** Summarizes patient demographics and RDQ scores.

Group	GERD group	rGERD group		*p*-value
	NERD	Refractory NERD	Refractory RE	
Number of cases (persons)	38	20	16	–
Male/female ratio (persons)	15/23	11/9	6/10	0.49
Age (years, $\bar{X}$ ± *s*)	50.69 ± 7.18	49.21 ± 10.43	55.60 ± 13.70	0.197
BMI (Kg/m^2^, $\bar{X}$ ± *s*)	22.44 ± 2.58	22.20 ± 2.07	22.27 ± 2.25	0.92
RDQ	18.00 (14.25, 19.75)	17.50 (13.00, 22.75)	19.50 (15.75, 24.50)	0.881^a^
				0.351^b^

^a^*p*-value for the comparison of RDQ scores between patients in the Refractory NERD Group and the NERD Group; ^b^*p*-value for the comparison of RDQ scores between the Refractory NERD Group and the Refractory RE Group.

### Symptom presentation

**Figure 1:**
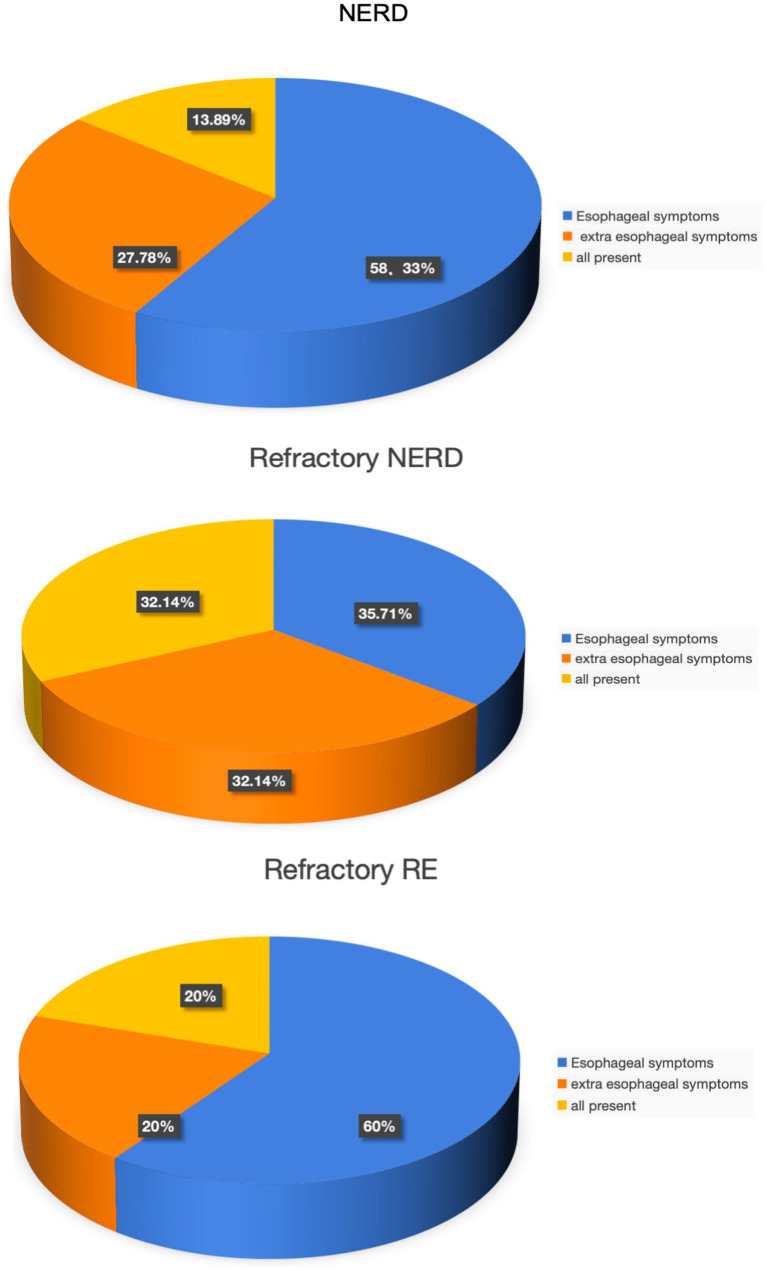
Distribution of reflux symptoms in each group.

Figure 1 reveals that among NERD (non-erosive reflux disease) patients, esophageal symptoms account for 58.33% of all symptomatic manifestations, while extra-esophageal symptoms represent 27.78%, and the co-occurrence of both types accounts for 13.89%. This indicates that NERD patients predominantly experience esophageal symptoms, primarily heartburn, acid regurgitation, and food regurgitation. In contrast, the refractory NERD group demonstrates a different pattern: esophageal symptoms constitute 35.71% of all symptoms, extra-esophageal symptoms account for 32.14%, and the simultaneous presence of both reaches 32.14%. While refractory NERD patients still frequently exhibit esophageal symptoms, there’s a notable increase in cases with extra-esophageal manifestations. This suggests that refractory NERD not only presents with typical acid reflux symptoms like heartburn and acid regurgitation but also often includes pharyngeal foreign body sensation and chronic cough. Regarding refractory RE (reflux esophagitis) patients, esophageal symptoms accounts for 60% of total symptoms, with extra-esophageal symptoms at 20.00% and their combination at 20.00%, demonstrating that this group also primarily manifests esophageal symptoms.

### Preoperative esophageal manometry results in refractory NERD vs. refractory RE

The refractory NERD group had a significantly higher mean resting pressure of the lower esophageal sphincter (LES) compared to the refractory RE group [13.05 (10.58, 16.85) mmHg vs. 6.15 (3.28, 11.43) mmHg; *z* = 1.706, *p* < 0.05]. Another pressure-related parameter, the distal contractile integral (DCI), was also higher in the refractory NERD group [813.20 (721.45, 894.18) mmHg·cm·s] than in the refractory RE group [679.00 (614.30, 715.53) mmHg·cm·s], with a statistically significant difference (p < 0.05). However, no significant differences were observed between the two groups in LES length (3.11 ± 0.52 cm vs. 2.84 ± 0.30 cm), integrated relaxation pressure (IRP) [7.15 (2.95, 13.38) mmHg vs. 7.00 (6.13, 10.70) mmHg], or contractile front velocity (CFV) [2.84 (2.35, 3.28) cm/s vs. 2.64 (2.43, 3.28) cm/s] (*p* > 0.05 for all). See Table 2 for details.

**Table 2:** Esophageal motility in refractory NERD versus refractory RE.

Parameter	LES length (cm)	LES resting pressure (mmHg)	DCI (mmHg·cm·s)	CFV (cm/s)	IBP (mmHg)
Refractory NERD	3.11 ± 0.52	13.05 (10.58, 16.85)	813.20 (721.45, 894.18)	2.84 (2.35 3.28)	7.15 (2.95 13.38)
Refractory RE	2.84 ± 0.30	6.15 (3.28, 11.43)	679.00 (614.30, 715.53)	2.64 (2.43, 3.28)	7.00 (6.13, 10.70)
Statistical index	*t* = 1.524	*z* = 1.706	*z* = −2.884	*z* = −0.696	*z* = −0.083
*p*-value	0.136	0.006	0.004	0.486	0.934

### Esophageal manometry findings in refractory NERD versus NERD

The refractory NERD group exhibited significantly lower mean resting pressure of the lower esophageal sphincter (LES) compared to the NERD group [13.05 (10.58, 16.85) mmHg vs. 15.70 (11.70, 22.78) mmHg; *z* = −2.153, *p* < 0.05]. Contractile front velocity (CFV) was significantly higher in refractory NERD patients [2.84 (2.35, 3.28) cm/s] than in NERD patients [2.42 (2.00, 2.74) cm/s; *z* = −2.734, p < 0.05]. However, no statistically significant differences were observed between the two groups in LES length (3.11 ± 0.52 cm vs. 3.35 ± 0.46 cm), distal contractile integral (DCI) [813.20 (721.45, 894.18) mmHg·cm·s vs. 844.20 (675.30, 1051.75) mmHg·cm·s], or integrated relaxation pressure (IRP) [7.15 (2.95, 13.38) mmHg vs. 7.90 (4.20, 12.43) mmHg]. See Table 3 for details.

**Table 3:** Esophageal motility in refractory NERD versus refractory.

Parameter	LES length (cm)	LES resting pressure (mmHg)	DC I (mmHg·cm·s)	CFV (cm/s)	IBP (mmHg)
Refractory NERD	3.11 ± 0.52	13.05 (10.58, 16.85)	813.20 (721.45, 894.18)	2.84 (2.35, 3.28)	7.15 (2.95 13.38)
Refractory RE	3.35 ± 0.46	15.70 (11.70, 22.78)	844.20 (675.30, 1051.75)	2.42 (2.00, 2.74)	7.90 (4.20, 12.43)
Statistical index	*t* = 1.568	*z* = −2.153	*z* = −1.089	*z* = −2.734	*z* = −0.521
*p*-value	0.122	0.031	0.276	0.006	0.602

### 24-h pH-impedance monitoring results in refractory NERD versus refractory RE groups

The refractory NERD group demonstrated significantly lower acid exposure time (13.30% [10.03, 17.73%]) compared to the refractory RE group (17.25% [12.95, 21.80%], *p* < 0.05). Similarly, the number of acid reflux episodes was significantly reduced in refractory NERD patients (19.50 [12.25, 45.50]) versus refractory RE patients (49.00 [33.75, 85.00], *p* < 0.05). The Demeester score was also significantly lower in the refractory NERD group (18.95 [16.10, 22.33]) than in the refractory RE group (31.25 [24.70, 43.35], p < 0.05). Conversely, the refractory NERD group showed significantly higher numbers of mixed gas–liquid reflux episodes (26.50 [20.25, 37.75]) and weakly acidic reflux episodes (29.50 [23.00, 40.50]) compared to the refractory RE group (19.50 [16.25, 25.25] and 19.50 [18.00, 26.25] respectively, both p < 0.05). However, no significant difference was observed in non-acid reflux episodes between refractory NERD (10.50 [8.00, 14.75]) and refractory RE groups (9.50 [6.50, 13.25], *p* > 0.05). See Table 4 for detailed results.

**Table 4:** 24-h pH-impedance monitoring: refractory NERD vs. RE.

Parameter	Acid exposure duration percentage (%)	Acid exposure episodes	Demeester score	Air–liquid mixed reflux episodes	Weak acid reflux episodes	Non-acid reflux episodes
Refractory NERD	13.30 (10.03, 17.73)	19.50 (12.25, 45.50)	18.95 (16.10, 22.33)	26.50 (20.25, 37.75)	29.50 (23.00, 40.50)	10.50 (8.00, 14.75)
Refractory RE	17.25 (12.95, 21.80)	49.00 (33.75, 85.00)	31.25 (24.70, 43.35)	19.50 (16.25, 25.25)	18.50 (18.00, 26.25)	9.50(6.50, 13.25)
Statistical index	*z* = −2.039	*z* = −2.836	*z* = −3.315	*z* = −2.073	*z* = −2.655	*z* = −0.798
*p*-value	0.041	0.005	0.001	0.038	0.008	0.425

### 24-h pH-impedance monitoring results in refractory NERD versus NERD groups

The refractory NERD group showed numerically lower but statistically non-significant values compared to the NERD group in acid exposure time (13.30% [10.03, 17.73%] vs. 13.55% [7.75, 18.47%]), number of acid reflux episodes (19.50 [12.25, 45.50] vs. 27.50 [21.50, 36.50]), and Demeester score (18.95 [16.10, 22.33] vs. 23.55 [15.30, 28.20]) (all *p* > 0.05). However, the refractory NERD group demonstrated significantly higher numbers of mixed gas–liquid reflux episodes (26.50 [20.25, 37.75] vs. 14.00 [11.00, 22.75]) and weakly acidic reflux episodes (29.50 [23.00, 40.50] vs. 23.00 [18.25, 27.00]) (both *p* < 0.05). No significant difference was observed in non-acid reflux episodes between refractory NERD (10.50 [8.00, 14.75]) and NERD groups (4.00 [8.50, 13.00], p > 0.05). See Table 5 for details.

**Table 5:** 24-h pH-impedance monitoring: Refractory NERD vs. NERD.

Parameter	Acid exposure duration percentage (%)	Acid exposure episodes	Demeester score	Air–liquid mixed reflux episodes	Weak acid reflux episodes	Non-acid reflux episodes
Refractory NERD	13.30 (10.03, 17.73)	19.50 (12.25, 45.50)	18.95 (16.10, 22.33)	26.50 (20.25, 37.75)	29.50 (23.00, 40.50)	10.50 (8.00, 14.75)
Refractory RE	13.55 (7.75, 18.47)	27.50 (21.50, 36.50)	23.55 (15.30, 28.20)	14.00 (11.00, 22.75)	23.00 (18.25, 27.00)	4.00 (8.50, 13.00)
Statistical Index	*z* = −0.379	*z* = −1.774	*z* = −1.665	*z* = −4.320	*z* = −2.920	*z* = −1.715
*p*-value	0.705	0.076	0.096	0	0.003	0.086

### Results of RSI and GERD-Q scoring

Statistical analysis of RSI and GERD-Q questionnaire scores obtained from each patient before admission and 6 months postoperatively revealed significant improvements. The postoperative scores at 6 months were 5.00 ± 2.05 points for RSI and 7.6 ± 1.5 points for GERD-Q, demonstrating statistically significant differences compared to preoperative values (all p < 0.05). Detailed results are presented in Table 6.

**Table 6:** Comparison of RSI and GERD-Q scores before and after surgery (*n* = 20, mean ± SD).

Time	RSI score	GERD-Q score
Preoperative	14.60 ± 6.95	14.0 ± 1.33
Six months after operation	5.00 ± 2.05	7.6 ± 1.5
*t*	6.05	10.67
*p*	0.00	0.00

### Histopathological manifestations in the study groups

**Figure 2:**
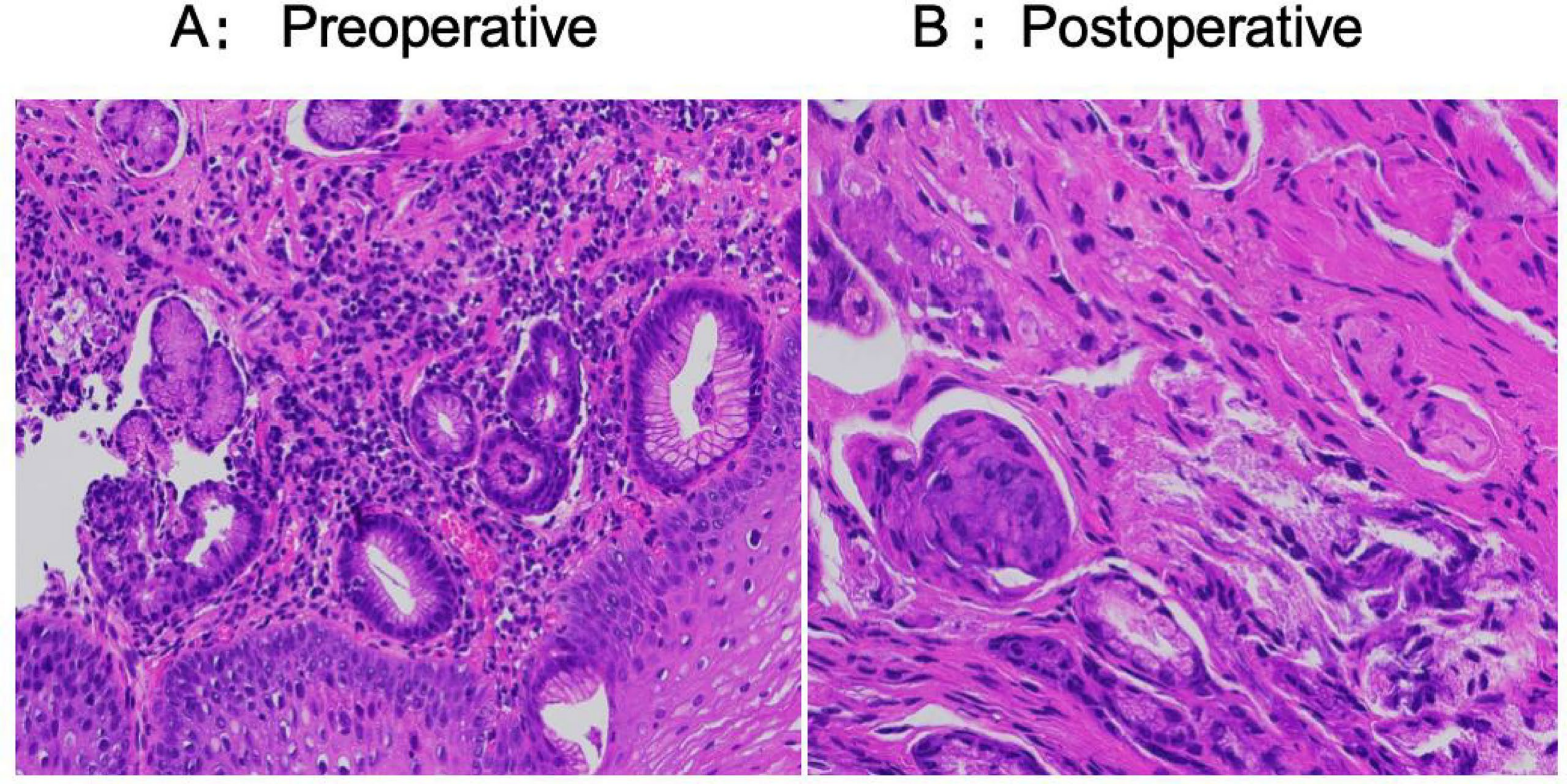
Histological analysis of esophageal mucosa before and after radiofrequency ablation treatment. **(A)** Preoperative biopsy showing preserved mucosal architecture with characteristic ‘U’-shaped glandular crypts, intact brush borders (BB), and abundant columnar cells (CC). Note significant inflammatory infiltrates (IF) predominantly composed of plasma cells and lymphocytes. **(B)** Postoperative specimen demonstrating marked reduction in inflammatory cell infiltration compared to preoperative findings (HE staining, ×400).

Endoscopic biopsy forceps were used to obtain one mucosal tissue sample from the esophageal lesion or approximately 2 cm above the dentate line. The specimen was immediately fixed in 10% formalin solution and subsequently processed through paraffin embedding, sectioning (4 μm thickness), slide mounting, labeling, and baking. Following the previously described HE staining protocol, the esophageal mucosal tissue was stained with hematoxylin and eosin (Figure 2). Histopathological analysis revealed that preoperative specimens maintained relatively intact mucosal architecture with characteristic “U”-shaped glandular crypts and preserved brush borders, showing numerous columnar cells alongside significant inflammatory infiltrates predominantly composed of plasma cells and lymphocytes. In contrast, postoperative specimens demonstrated markedly reduced inflammatory cell infiltration compared to preoperative findings.

### Expression of PAR2 in esophageal tissues across study groups

#### Immunohistochemical results of PAR2

**Figure 3:**
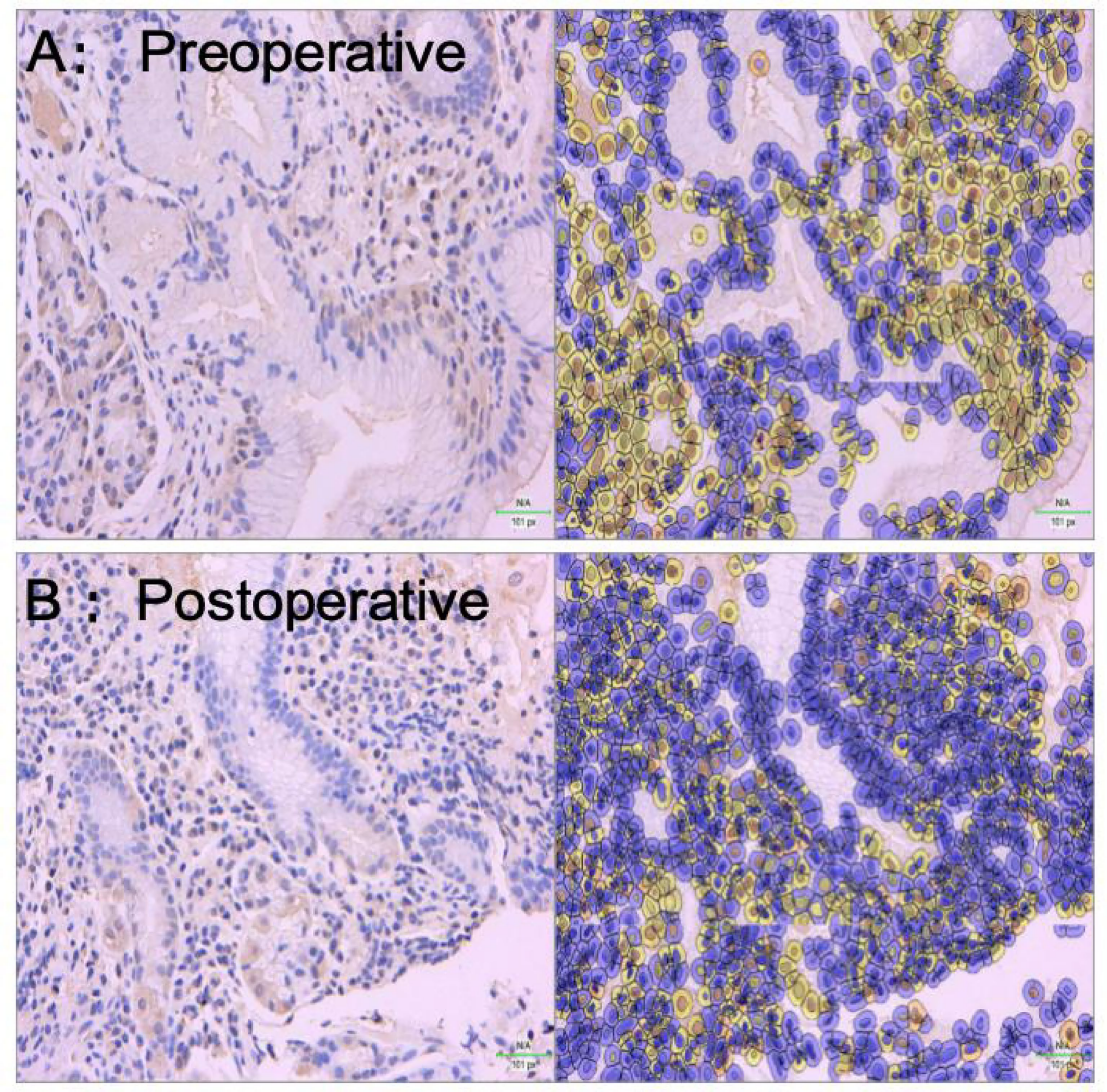
Immunohistochemical analysis of PAR2 expression in esophageal tissues. **(A)** Preoperative specimen showing strong PAR2 immunoreactivity primarily localized in the lamina propria (LP), mucosal layer (ML), and submucosa (SM). **(B)** Postoperative specimen exhibiting significantly weaker and more sparse PAR2 staining compared to preoperative levels. The postoperative specimen demonstrates reduced PAR2 expression in the same anatomical regions (IHC staining, ×400).

**Figure 4:**
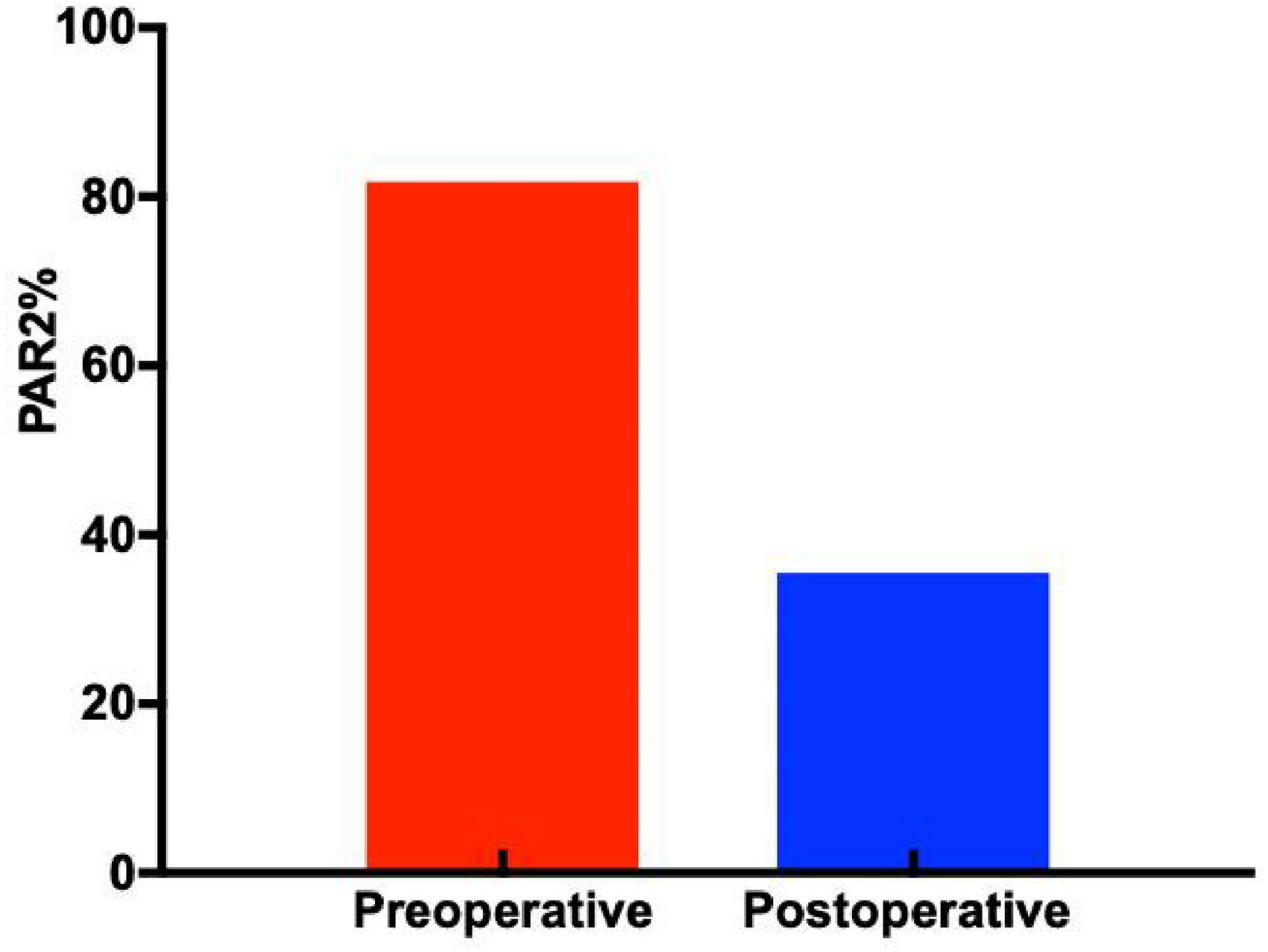
Expression of PAR2 positive cells in each group (*p* < 0.05). Western blot analysis of PAR2 expression.

PAR2 positive staining was observed in most specimens, primarily localized in the lamina propria, mucosal layer, and submucosa. Compared to preoperative samples, postoperative specimens exhibited significantly weaker and more sparse PAR2 immunostaining (Figure 3). Quantitative analysis revealed PAR2 expression levels of 81.8 ± 14.9% in preoperative tissues versus 35.5 ± 5.8% in postoperative tissues, demonstrating a statistically significant difference (*p* < 0.05, Figure 4).

**Figure 5:**
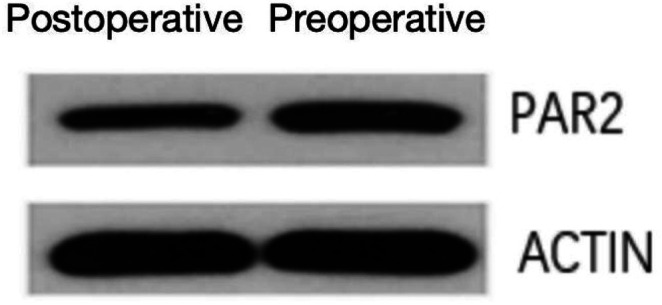
Western blot analysis of PAR2 expression in esophageal tissues across experimental groups.

**Figure 6:**
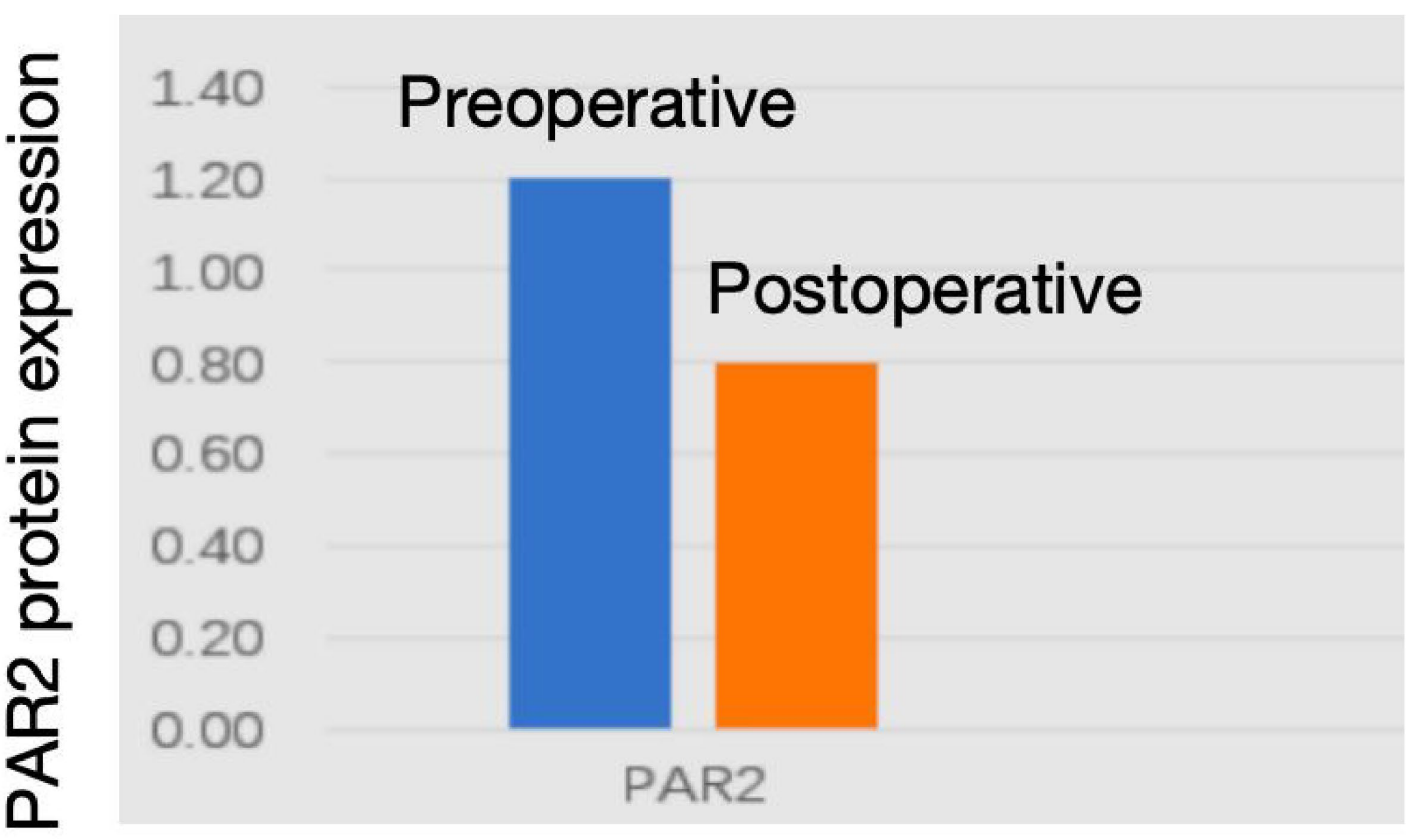
Quantitative analysis of western blot results (*p* < 0.05).

Western blot analysis revealed significantly lower PAR2 protein expression levels in postoperative esophageal mucosa compared to preoperative samples (0.8 ± 0.2 vs. 1.2 ± 0.3, *p* < 0.05), with corresponding reductions in PAR2 mRNA levels (Figures 5, 6).

### Expression of TRPV1 in esophageal tissues across study groups

#### IHC findings of TRPV1 expression

**Figure 7:**
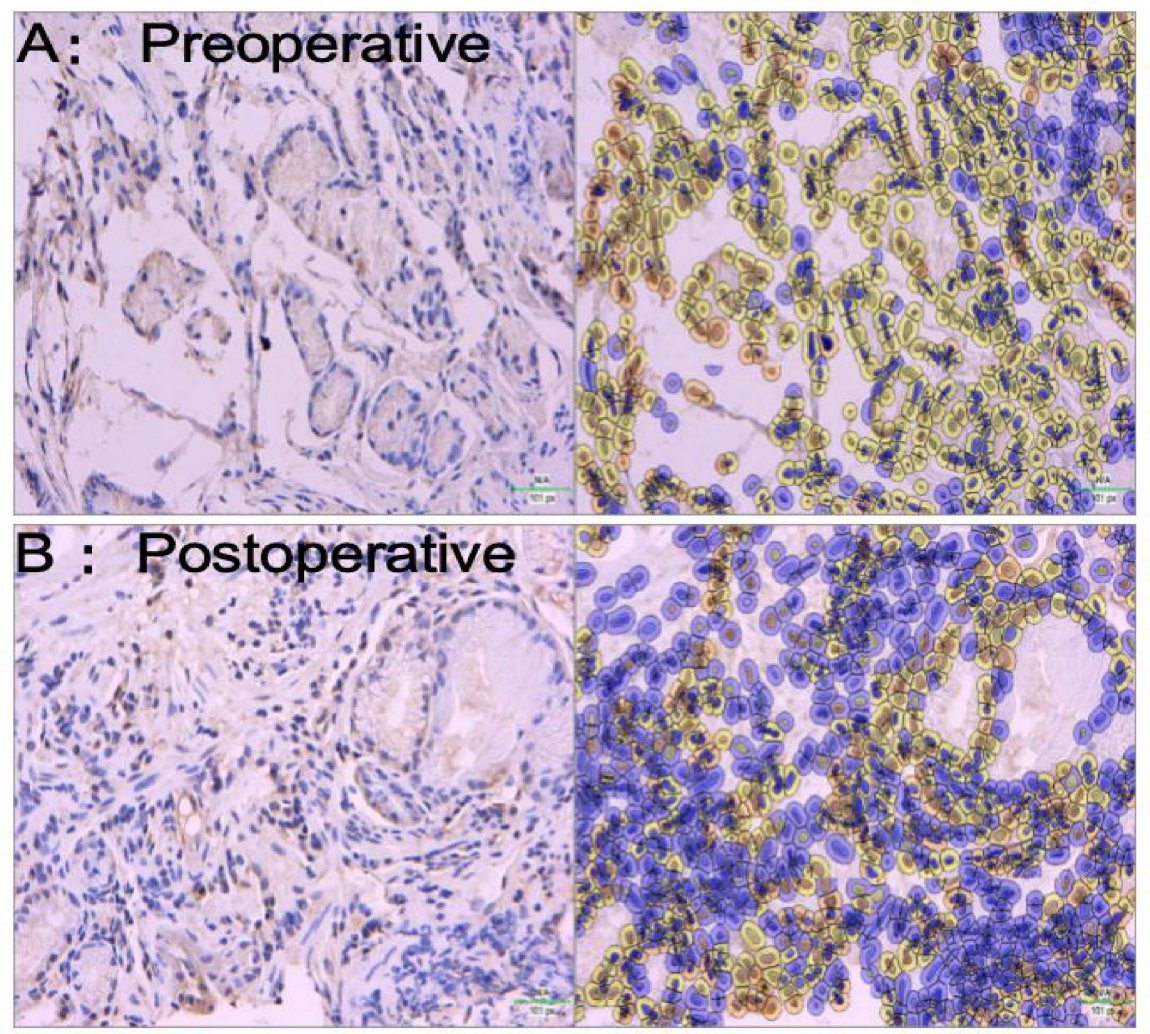
Representative immunohistochemical images of TRPV1 expression in esophageal tissues across patient groups (IHC, ×400). **(A)** Preoperative specimen showing characteristic TRPV1 immunoreactivity distributed throughout the esophageal wall structure. **(B)** Postoperative specimen demonstrating significantly attenuated and more sparse TRPV1 staining compared to preoperative levels, indicating reduced esophageal hypersensitivity following radiofrequency ablation therapy.

**Figure 8:**
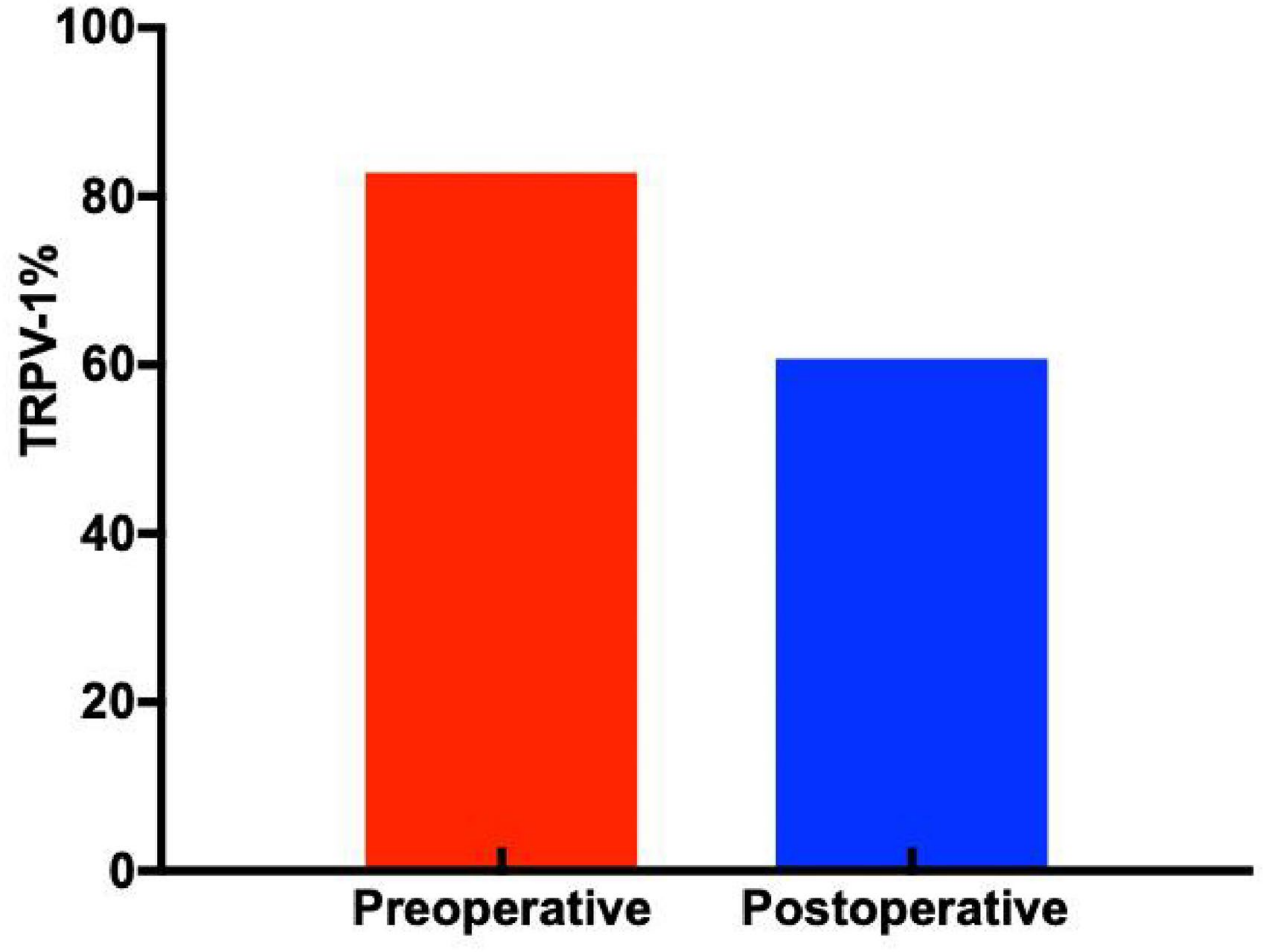
Quantification of TRPV1-positive cells across study groups (*p* < 0.05).

TRPV1 immunopositive cells were observed in the majority of specimens, primarily localized within the lamina propria, mucosal layer, and submucosa. Comparative analysis revealed that postoperative samples exhibited significantly weaker and more sparse TRPV1 immunostaining compared to preoperative specimens. Representative immunohistochemical images of esophageal tissues from each patient group are presented in Figure 7. Quantitative analysis demonstrated statistically significant differences in TRPV1-positive cell expression levels among the groups (*p* < 0.05), as detailed in Figure 8.

#### Western blot analysis of TRPV1 expression

**Figure 9:**
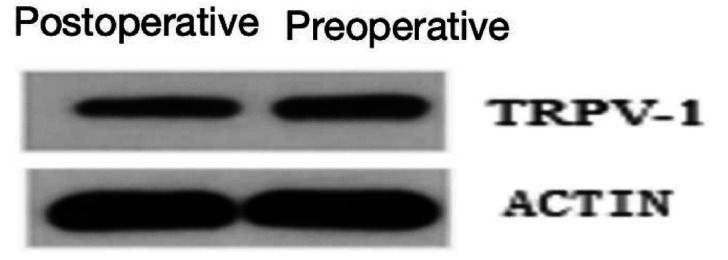
Protein expression of TRPV1 in esophageal tissues across experimental groups.

**Figure 10:**
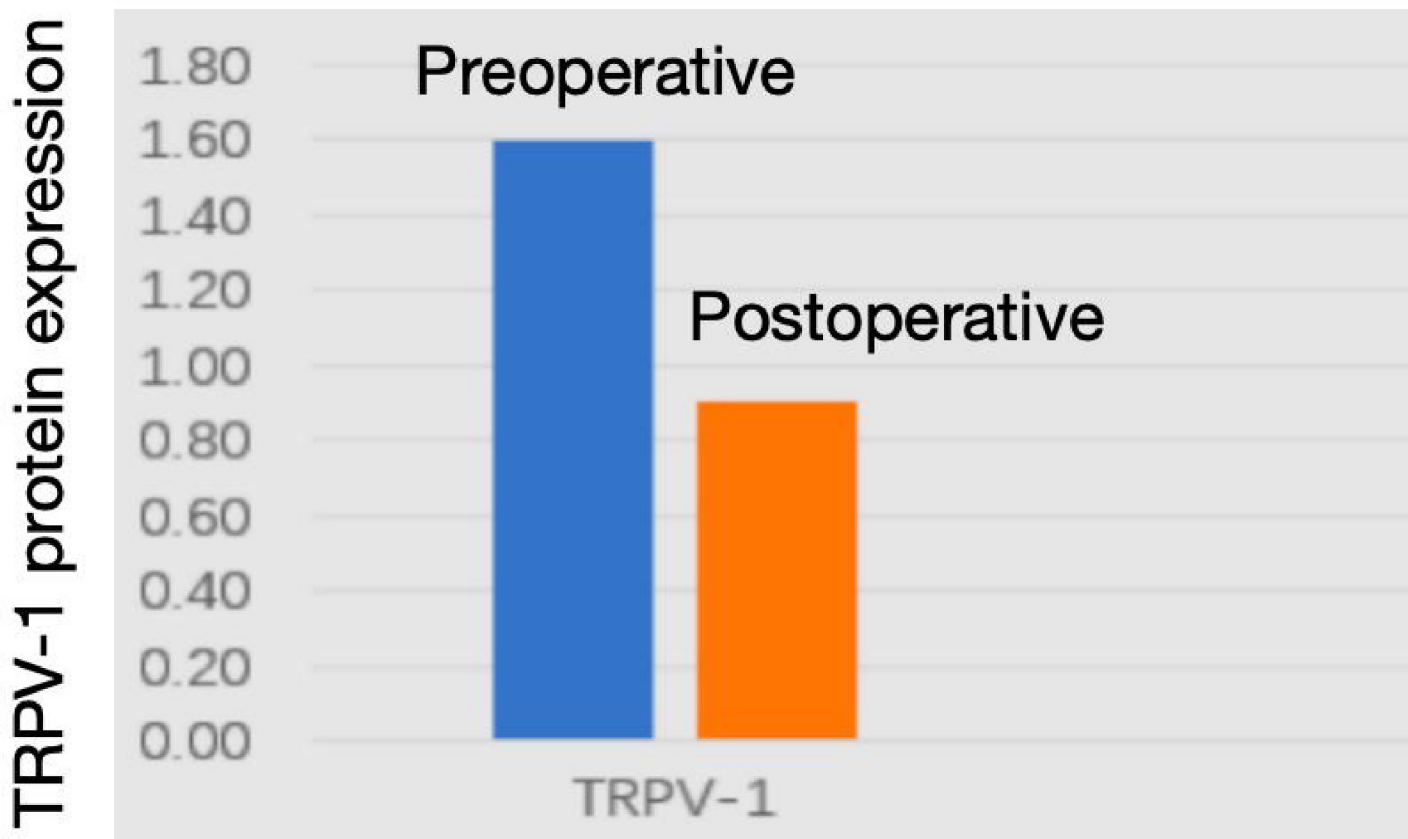
Quantified western blot results (*p* < 0.05).

Western blot analysis demonstrated significantly reduced TRPV1 mRNA levels in postoperative esophageal mucosa compared to preoperative samples. Quantitative protein analysis revealed TRPV1 expression levels of 1.6 ± 0.19 in preoperative tissues versus 0.9 ± 0.2 in postoperative specimens, showing statistically significant differences (*p* < 0.05, Figures 9, 10).

#### Expression of PAR2 in serum specimens across study groups

**Figure 11:**
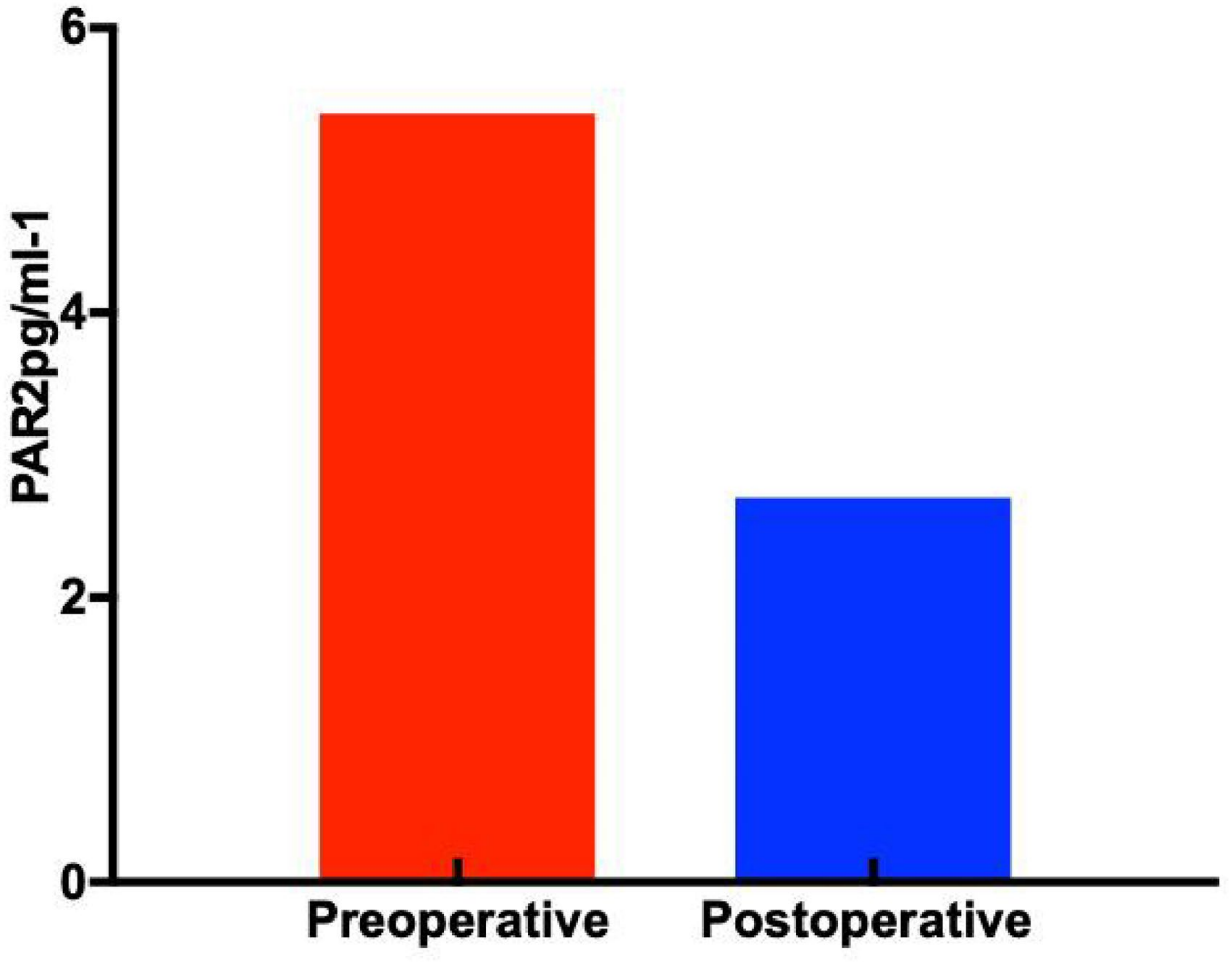
Expression of PAR2 in serum samples (*p* < 0.05).

ELISA results for PAR2 demonstrated significantly higher expression levels in patients with GERD preoperatively (5.4 ± 2.3 pg./mL) compared to postoperative measurements (2.7 ± 0.9 pg./mL), with a statistically significant difference (p < 0.05, Figure 11).

#### Expression of TRPV1 in serum samples across study groups

**Figure 12:**
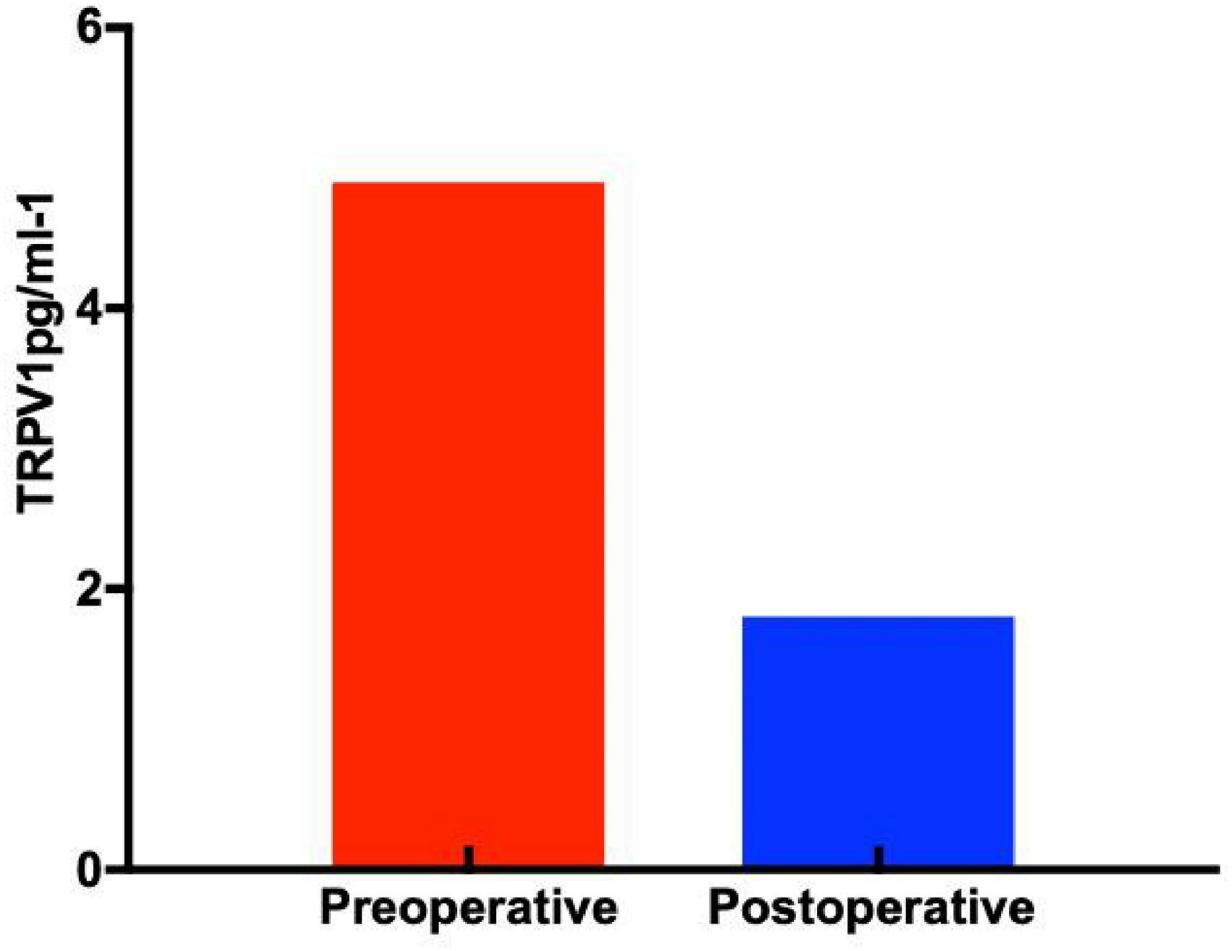
Expression of TRPV1 in serum samples (*p* < 0.05).

ELISA results for TRPV1 revealed significantly higher preoperative expression levels in patients with GERD (4.9 ± 4.1 pg./mL) compared to postoperative measurements (1.8 ± 1.2 pg./mL), with a statistically significant difference (p < 0.05, Figure 12).

## Discussion

With societal development and continuous changes in living standards and lifestyles, the annual incidence rate of rGERD has shown a significant upward trend (24–26), making GERD one of the most prevalent chronic disorders in the digestive system. Studies indicate that the occurrence of rGERD correlates with dietary and lifestyle habits, including consumption of sweets, high-fat diets, spicy foods, alcohol intake, and smoking, as well as factors like obesity and constipation (27). The accelerated pace of modern life has also contributed to this condition, as stress, anxiety, and tension among younger populations may predispose them to GERD. Research further demonstrates that anxiety and depression are prevalent in some rGERD patients, potentially facilitating the progression from GERD to rGERD. In such cases, treating only the somatic symptoms may yield suboptimal clinical outcomes, and adjunctive therapy with anxiolytic or antidepressant medications might be necessary for rGERD patients with significant psychological comorbidities (28). Many individuals experience varying degrees of gastroesophageal reflux symptoms in daily life, with acid regurgitation and heartburn being the most common typical manifestations. While proton pump inhibitor (PPI) therapy often effectively alleviates these classic GERD symptoms, studies have found that rGERD patients—particularly those with NERD—frequently present with atypical and extra-esophageal symptoms (29), including chest pain, globus pharyngeus, chronic cough, and asthma-like symptoms. In our study, no significant differences were observed in gender, age, BMI, or RDQ scores among the NERD, refractory NERD, and refractory RE groups. The NERD group predominantly exhibited esophageal symptoms (acid regurgitation, heartburn, food reflux), whereas refractory NERD patients demonstrated a relative decrease in esophageal symptoms alongside increased extra-esophageal/atypical manifestations (chest pain, throat irritation, cough, asthma, bloating, belching). These findings indicate that although both NERD and refractory NERD cohorts predominantly manifest esophageal symptoms, the latter group shows more pronounced extra-esophageal involvement. In contrast, refractory RE patients mainly presented with esophageal symptoms. The pathophysiology of extra-esophageal symptoms remains incompletely understood but may involve persistent low-level acid or non-acid reflux events.

With the rising incidence of GERD, research on esophageal motility has progressively advanced, leading to a growing consensus among scholars that GERD results from the combined effects of various motility disorders (30, 31). However, studies examining esophageal motility characteristics in rGERD patients remain relatively scarce in both domestic and international literature. HRM offers distinct advantages for such investigations: it enables dynamic observation of LES pressure variations to assess anti-reflux barrier function and directly identify esophageal motility abnormalities; it captures the dynamic movement processes of esophageal sphincters and body, including esophagogastric junction function and peristaltic patterns; and it facilitates differential diagnosis of conditions like achalasia and functional obstruction (32). Current research indicates that GERD-related motility disorders typically involve several key mechanisms: hypotensive or absent LES pressure with transient LES relaxations (TLESRs); impaired esophageal clearance due to motility dysfunction; alterations in the esophagogastric angle (Angle of His); compromised mucosal barrier function; changes in cardia mucosal function; and psychological factors (33). Among these, dysfunction of the esophageal anti-reflux barrier—manifested primarily through transient LES relaxations, reduced LES pressure, and ineffective esophageal motility—constitutes one of the critical pathophysiological mechanisms in GERD (34). Notably, decreased LES resting pressure and shortened LES length have been associated with poorer treatment outcomes in rGERD and increased susceptibility to symptom recurrence (35). In this study, the refractory RE group exhibited LES resting pressures below normal reference values, while the refractory NERD group showed pressures at the lower limit of normal range—both significantly lower than the NERD group, suggesting that reduced LES pressure likely plays a pivotal role in GERD pathogenesis. Notably, the refractory NERD group demonstrated significantly shorter LES lengths compared to NERD patients, further underscoring the importance of LES integrity in refractory cases. The distal contractile integral (DCI), a pressure-derived metric quantifying esophageal contraction vigor, revealed that the refractory RE group had significantly lower DCI values than refractory NERD patients, potentially reflecting weakened esophageal body contractions that impair effective refluxate clearance, thereby exacerbating symptom severity. Effective peristalsis is critical for bolus transit and timely clearance of refluxed material; diminished motility prolongs esophageal exposure to noxious stimuli, aggravating mucosal injury and symptoms like heartburn. However, the absence of DCI differences between refractory and non-refractory NERD groups implies that contraction amplitude may not contribute to NERD refractoriness. Intriguingly, higher contractile front velocities (CFV) were observed in rGERD patients—particularly refractory NERD—compared to both refractory RE and non-refractory NERD groups, despite all values remaining within normal limits (<10 cm/s). While slower CFV typically correlates with prolonged esophageal clearance times and mucosal damage (36, 37), the elevated CFV in refractory NERD suggests alternative pathophysiological mechanisms unrelated to peristaltic speed. Emerging evidence implicates genetic predisposition and esophageal hypersensitivity in rGERD (38), and our findings support the hypothesis that additional factors beyond conventional motility defects—particularly in refractory NERD—may drive PPI treatment failure, warranting further investigation.

The 24-h multichannel intraluminal impedance-pH (MII-pH) monitoring serves as a critical diagnostic tool for identifying pathological gastroesophageal reflux of various properties, capable of detecting reflux events with pH below 4 through pH above 7 and differentiating their physical states. Refluxate was categorized based on pH thresholds: acid reflux (pH < 4), weakly acidic reflux (pH 4–7), and non-acid reflux (pH > 7) (39). While traditional GERD primarily involves acid reflux (pH < 4), driving classic symptoms like heartburn (40), studies reveal that 50% of NERD patients exhibit no acid/non-acid reflux but demonstrate acid hypersensitivity with altered visceral perception (41). Emerging evidence identifies weakly acidic reflux and esophageal hypersensitivity as major contributors to GERD treatment failure (42). Our comparative analysis of refractory NERD, refractory RE, and NERD groups revealed that refractory NERD patients had fewer acid exposure episodes/shorter duration than refractory RE patients—factors known to exacerbate mucosal injury—but exhibited more frequent weakly acidic reflux events. This suggests incomplete PPI suppression of weakly acidic reflux may underlie rGERD pathogenesis, particularly in refractory NERD where such reflux proves more resistant to PPI therapy compared to acid-dominated reflux esophagitis. Although PPIs remain first-line GERD therapy, their limited efficacy in some patients may stem from weakly acidic reflux and proximal reflux patterns that evade pharmacological suppression, facilitating progression to rGERD. Boeckxstaens et al.’s meta-analysis documented a 43% increase in weakly acidic reflux among PPI-naïve patients with GERD versus those on standard PPI regimens (43). Post-PPI treatment, while acid exposure improves, gas–liquid mixed and non-acid reflux often persist (44), aligning with our findings of elevated mixed reflux episodes in refractory NERD versus both refractory RE and NERD groups. The larger volume of gas–liquid refluxates may intensify symptom perception, while increased weakly acidic/non-acid reflux events—more prevalent in refractory NERD—likely drive atypical/extra-esophageal symptoms (45). Clinical studies note that rGERD symptoms predominantly manifest atypically (46), with weak-acid reflux showing stronger correlation with such symptoms than acid reflux (45). PPIs effectively alleviate typical symptoms but demonstrate poor response against weakly acidic/non-acid reflux-related atypical symptoms (47, 48). MII-pH data further distinguish rGERD from GERD through pH profiles (more weakly acidic/non-acid reflux) and impedance patterns (more non-liquid reflux), with refractory NERD exhibiting the highest frequencies of these reflux subtypes, highlighting their pathogenic significance.

The aforementioned findings demonstrate that rGERD patients, particularly those with refractory NERD, exhibit more prominent atypical and extra-esophageal symptoms. Refractory NERD patients display distinct esophageal motility abnormalities compared to both NERD and refractory RE patients, characterized by a higher prevalence of weakly acidic and gas–liquid mixed reflux events. To investigate whether these phenomena are associated with esophageal hypersensitivity in rGERD patients, we selected 20 enrolled rGERD cases for endoscopic therapy, systematically evaluating changes in clinical symptoms and expression levels of hypersensitivity receptors PAR2 and TRPV1 before and after the procedure.

TRPV1 was initially identified in primary sensory neurons as the receptor for capsaicin and related vanilloid compounds (49). It is expressed in sensory nerves and closely associated with the expression of nerve growth factor (NGF) and glial cell line-derived neurotrophic factor (GDNF). Patients with esophagitis demonstrate significantly elevated mucosal levels of NGF and GDNF mRNA compared to asymptomatic individuals and healthy controls (50). Current evidence suggests TRPV1 serves as a molecular integrator of inflammatory responses to noxious stimuli (including acid) and is expressed at esophageal mucosal levels. To confirm TRPV1 mRNA presence in non-neuronal cells, enzymatically isolated and flow-sorted esophageal epithelial cells from acid-induced esophagitis animal models showed substantial TRPV1 expression (both mRNA and protein) (51). Furthermore, acid-induced esophagitis was markedly attenuated in TRPV1-deficient animals or through pharmacological interventions like acid suppressants (famotidine/omeprazole) or receptor antagonists in wild-type mice (52). Two recent human studies of esophageal mucosal biopsies revealed significantly higher TRPV1 mRNA and protein expression in patients with GERD—particularly those with erosive esophagitis—versus asymptomatic patients and healthy controls (53, 54). Upregulated TRPV1 in inflamed esophageal mucosa from both animal models and esophagitis patients implies its potential role in modulating inflammatory responses beyond nociception. Indeed, TRPV1 activation by acid in esophageal mucosa triggers release of inflammatory mediators including substance P (SP), calcitonin gene-related peptide (CGRP), and platelet-activating factor (PAF). The expression of PAF, SP, and CGRP was inhibited by selective TRPV1 antagonist 5′-iodoresiniferatoxin, while neurotoxin blocker tetrodotoxin suppressed only SP and CGRP but not PAF (55), suggesting TRPV1 induces PAF through neuron-independent pathways. As a key inflammatory mediator, PAF acts as a chemoattractant for immune cells and—via subsequent release of IL-8, IL-1, and IL-6—reduces esophageal muscle contractions, thereby promoting gastroesophageal reflux. Animal studies demonstrate TRPV1’s greater impact on acid perfusion-induced muscle contraction compared to ASIC3, another acid-sensing receptor family member (56). Notably, acid-exposed esophageal epithelial cells exhibit PAR2 overexpression, indicating its critical role in GERD pathogenesis. Our experimental data confirm the presence of both TRPV1 and PAR2 acid-sensing receptors in the esophageal mucosa of patients with GERD.

Given the role of esophageal sensitivity in GERD symptom manifestation, reduced esophageal sensitivity may contribute to symptom improvement. Esophageal acid sensitivity depends on multiple factors, including hydrogen ion exposure, mucosal permeability, the quantity and activation state of acid-sensitive nerve endings, and central processing of afferent sensory signals. Esophageal inflammation is generally considered a contributing factor to increased acid sensitivity. Notably, accumulating evidence demonstrates that Stretta therapy can achieve healing of erosions. A study by Liu et al. (57) demonstrated that the rate of PPI use among treated patients decreased from 100% at baseline to 76.7% at 12 months, with some patients discontinuing medication or using it only as needed. Corresponding improvements in endoscopic grading of esophagitis were observed in 33 of 41 patients. By 6 months, all patients either showed no erosions or only mild grade A erosions. Histopathological analysis in our study revealed relatively intact preoperative esophageal mucosal architecture, characterized by “U”-shaped glandular crypts, preserved brush borders, and numerous columnar cells, alongside significant inflammatory infiltrates predominantly composed of plasma cells and lymphocytes. Postoperative specimens demonstrated markedly reduced inflammatory cell infiltration compared to preoperative findings. The mechanism of endoscopic GERD treatment primarily involves narrowing the cardia diameter, the significant symptom relief observed in most patients post-procedure raises the question of whether this improvement stems solely from anatomical changes or involves additional factors. Our research team hypothesizes that postoperative symptom alleviation may be associated with modifications in esophageal sensitivity.

While previous studies have confirmed upregulated TRPV1 and PAR2 expression in the esophageal mucosa of patients with GERD, existing literature has mainly focused on expression differences of these receptors across different GERD subtypes (NERD, RE). Our study extends this knowledge by providing systematic evidence that endoscopic radiofrequency ablation (RFA) can effectively modulate the expression of these hypersensitivity receptors in patients with refractory non-erosive reflux disease (rNERD). Our experimental results demonstrate that immunohistochemistry, Western blot, and ELISA analyses consistently revealed reduced expression of esophageal sensitivity receptors TRPV1 and PAR2 postoperatively compared to preoperative levels. Concurrently, postoperative patients showed decreased RSI and GERD-Q scores, and histological examination via HE staining indicated attenuated inflammatory cell infiltration in esophageal mucosal tissues following treatment. These findings collectively suggest that endoscopic therapy can downregulate the expression of esophageal sensitivity receptors, with receptor expression levels positively correlating with both clinical symptom scores and the degree of inflammatory cell infiltration. However, the precise mechanisms through which endoscopic treatment reduces TRPV1 and PAR2 receptor expression in patients with GERD remain unclear and warrant further investigation. A key methodological advance of this study lies in its integrated assessment approach. Previous investigations have typically examined either receptor expression patterns OR structural improvements post-treatment. Our research bridges this divide by evaluating both molecular and clinical parameters concurrently. The originality of our work does not lie in identifying these receptors in GERD pathophysiology—this has been established in prior studies (19, 54). Rather, our unique contribution is demonstrating that endoscopic intervention can actively regulate these established pathways.

This study has several limitations that should be considered when interpreting the findings. First, the retrospective, observational design precludes definitive causal inferences between RFA and the observed reductions in esophageal hypersensitivity markers. The absence of a control group (e.g., patients receiving optimized medical therapy, another endoscopic modality, or a sham procedure) limits our ability to attribute the observed effects solely to RFA and to rule out potential influences from placebo effects or regression to the mean. However, to mitigate these limitations, we implemented blinded assessment of all histological and molecular endpoints and employed within-subject pre-post comparisons, which control for inter-individual baseline variability. Second, the modest sample size (*n* = 20), while providing sufficient power to detect large effect sizes for our primary molecular outcomes as noted in the Methods, restricts the statistical power for more complex subgroup analyses and increases the risk of type II errors. Finally, although we adjusted for key clinical covariates (age, sex, BMI, baseline symptom scores) in our analyses, unmeasured confounding factors cannot be entirely excluded. These limitations notwithstanding, our study provides compelling preliminary mechanistic insights. Future prospective, randomized controlled trials with larger cohorts and appropriate control groups are warranted to confirm these observations, establish causality, and further elucidate the therapeutic mechanisms of RFA in rNERD.

## Conclusion

Refractory NERD is characterized by distinct esophageal motility dysfunction compared to NERD and refractory RE, with weak acid and gas–liquid reflux likely contributing to esophageal hypersensitivity. Endoscopic treatment effectively alleviates clinical symptoms, improves histological damage, and reduces esophageal sensitivity. Symptom relief correlates positively with decreased expression of esophageal sensitivity receptors.

## Data Availability

The original contributions presented in the study are included in the article/supplementary material, further inquiries can be directed to the corresponding author.

## References

[1] Maret-Ouda J Markar SR Lagergren J. Gastroesophageal reflux disease: a review. JAMA. (2020) 324:2536–47. doi: 10.1001/jama.2020.21360, 33351048

[2] Vakil N van Zanten SV Kahrilas P Dent J Jones R Global CG. The Montreal definition and classification of gastroesophageal reflux disease: a global evidence-based consensus. Am J Gastroenterol. (2006) 101:1900–1920, quiz 43. doi: 10.1111/j.1572-0241.2006.00630.x 16928254

[3] Dent JA. Dew technique for continuous sphincter pressure mea- surement. Gastroenterology. (1976) 71:263–7. 939387

[4] Clouse RE Staiano A.Topography of the esophageal peristaltic pressure wave.Am J Phys, 1991, 261:G677–G684, doi: 10.1152/ajpgi.1991.261.4.G677, . 1928353

[5] Xiang G. Clinical diagnostic and therapeutic thinking of gastroesophageal reflux disease. Chin J Clin Phys. (2016) 44:3–5.

[6] Inadomi JM McIntyre L Bernard L Fendrick AM.Step-down from multiple-to single- dose proton pump inhibitors(PPIs):a prospective study of patients with heartburn or acid regurgitation completely relieved with PPIs.Am J Gastroenterol 2003:98:1940–1944, doi: 10.1111/j.1572-0241.2003.07665.x, . 14499769

[7] Gallup Organization. The 2000 Gallup study of consumers’ use of stomach relief products. Princeton: Gallup organization (2000).

[8] Chinese Society of Gastroenterology, Chinese Medical Association. 2014 Chinese expert consensus on gastroesophageal reflux disease. Chin J Dig. (2014) 10:649–61.

[9] Chen S. Etiological analysis and treatment strategies for refractory gastroesophageal reflux disease. World Latest Med Inf. (2016):103.

[10] Wang Y Gao H Yang X Yang Z. Clinical study on the adjunctive therapeutic effect of anti-anxiety and anti-depressant drugs in refractory gastroesophageal reflux disease. Chin J Pract Nerv Dis. (2013) 16:42–3.

[11] Liang Y Xu Y. Analysis of 24-hour impedance-pH monitoring in patients with refractory gastroesophageal reflux disease. Bethune Med J. (2016) 14:198–9.

[12] Wang K Duan L Xia Z Xu Z Ge Y. Esophageal motility characteristics in patients with refractory heartburn based on high-resolution esophageal manometry and impedance-pH monitoring. Natl Med J China. (2014) 94:2650–5. 25511590

[13] Li M Leng Y Guo Z Zhang C. Analysis and nursing care of 24-hour esophageal pH-impedance monitoring and high-resolution esophageal manometry in patients with refractory gastroesophageal reflux disease. Chin J Mod Nurs. (2017) 23:2645–7.

[14] Kessing BF Bredenoord AJ Weijenborg PW Hemmink GJ Loots CM Smout AJ. Esophageal acid exposure decreases intraluminal baseline impedance levels. Am J Gastroenterol. (2011) 106:2093–7. doi: 10.1038/ajg.2011.276, 21844921

[15] Kandulski A Weigt J Caro C Jechorek D Wex T Malfertheiner P. Esophageal intraluminal baseline impedance differentiates gastroesophageal reflux disease from functional heartburn. Clin Gastroenterol Hepatol. (2015) 13:1075–81. doi: 10.1016/j.cgh.2014.11.033, 25496815

[16] Liu B Yao S Zhang Y Wang M Du S Liu L. Correlation between esophageal impedance baseline, acid exposure, and epithelial intercellular space in patients with non-erosive reflux disease. Acad J Chin PLA Med Sch. (2015) 36:204–7.

[17] Seo AY Shin CM Kim N Yoon H Park YS Lee DH. Correlation between hypersensitivity induced by esophageal acid infusion and the baseline impedance level in patients with suspected gastroesophageal reflux. J Gastroenterol. (2015) 50:735–43. doi: 10.1007/s00535-014-1013-4, 25479939

[18] Kohata Y Fujiwara Y Yamagami H Tanigawa T Shiba M Watanabe K . Usefulness of baseline impedance in patients with proton pump inhibitor –refractory nonerosive reflux disease. J Gastroenterol Hepatol. (2015) 30:36–40. doi: 10.1111/jgh.12743, 25827802

[19] Abd ED Fath EH Kamal EM. Expression of proteinase-activated receptor-2 in the esophageal mucosa of gastroesophageal reflux disease patients: a histomorphologic and immunohistochemical study. Appl Immunohistochem Mol Morphol. (2015) 23:646–52. 25265427 10.1097/PAI.0000000000000130

[20] Barlow WJ Orlando RC. The pathogenesis of heartburn in nonerosive reflux disease: a unifying hypothesis. Gastroenterology. (2005) 128:771–8. doi: 10.1053/j.gastro.2004.08.014, 15765412

[21] Aziz Q Fass R Gyawali CP Miwa H Pandolfino JE Zerbib F. Functional esophageal disorders. Gastroenterology. (2016) 150:1368–79. doi: 10.1053/j.gastro.2016.02.012 27144625

[22] Ergun M Dogan I Unal S. Ineffective esophageal motility and gastroesophageal reflux disease: a close relationship? Turk J Gastroenterol. (2012) 23:627–33. doi: 10.4318/tjg.2012.0452, 23794296

[23] Herregods TV Bredenoord AJ Smout AJ. Pathophysiology of gastroesophageal reflux disease: new understanding in a new era. Neurogastroenterol Motil. (2015) 27:1202–13. doi: 10.1111/nmo.12611, 26053301

[24] Yang J Chen GH Liu M. Combiantion therapy 56 patients of proton pump inhibitor hydrotalcite and bailemian in refractory gastroesophageal reflux disease(GERD). Int J Dig Dis. (2012) 32:769–770.

[25] Wang H Li L Zhang H Xu G. Some study advances of diagnosis, treatment and pathosenes of GERD. Lab Med Clin. (2012) 9:1426–9.

[26] Tu L Hou X. Impact of lifestyle and dietary factors on gastroesophageal reflux. Chin J Intern Med. (2011) 50:636–9.

[27] Jiang L Ye B Lin L Zhang H Li X Wang M. Clinical characteristics analysis of outpatients with refractory gastroesophageal reflux disease. Global Trad Chin Med. (2013) S1:275–6.

[28] Yang D Suo Z Li F Hu J Yu L Zhao J . Therapeutic effect of flupentixol and melitracen in 140 cases of refractory gastroesophageal reflux disease with anxiety and depression. Chin J Dig. (2013) 33:476–8.

[29] Wang J Yuan Y Xu W Sun J. Clinical study on extra-esophageal symptoms of gastroesophageal reflux disease: laryngopharyngitis. Chin J Dig. (2006) 26:6–9.

[30] Valezi AC Herbella F Schlottmann F Patti MG. Gastroesophageal reflux disease in obese patients. J Laparoendosc Adv Surg Tech A. (2018) 28:949–52. doi: 10.1089/lap.2018.0395, 30004267

[31] Tang Y Qian A Zhu Y Yao W Huang J. Comparison of esophageal motility between refractory and non-refractory gastroesophageal reflux disease. J Intern Med Conc Pract. (2015) 10:418–20.

[32] Yang Jia Esophageal Motility Analysis in Patients with Refractory Gastroesophageal Reflux Disease and the Value of Different Swallowing Tests in Esophageal Manometry Zhejiang University, Master's thesis 2015.

[33] Biasutto D Roman S Garros A Mion F. Esophageal shortening after rapid drink test during esophageal high-resolution manometry: a relevant finding? United European Gastroenterol J. (2018) 6:1323–30. doi: 10.1177/2050640618796752, 30386605 PMC6206544

[34] Knigge MA Marvin S Thibeault SL. Safety and tolerability of pharyngeal high-resolution manometry. Am J Speech Lang Pathol. (2018) 28:43-52. doi: 10.1111/nmo.12745 30515521

[35] Yamashita H Okada A Naora K Hongoh M Kinoshita Y. Adding acotiamide to gastric acid inhibitors is effective for treating refractory symptoms in patients with non-erosive reflux disease. Dig Dis Sci. (2018) 64:823–31. doi: 10.1007/s10620-018-5377-9, 30465175 PMC6394577

[36] Li S Chen S Gao H. Investigation of reflux risk factors in refractory gastroesophageal reflux disease. Chin J Clin Med. (2013) 20:696–8.

[37] Zuo Z Pu X An Y Li Y Fan H Guo Q. Correlation analysis of lower esophageal sphincter structural and functional abnormalities in refractory GERD patients using endoscopic ultrasound and high-resolution manometry. Chin J Endosc. (2015) 21:914–8.

[38] Song H Jiang Y Zhang S You L Hu Y. Study on reflux events in patients with non-erosive reflux disease. J Clin Gastroenterol. (2016) 28:220–3.

[39] Patel A Sayuk GS Kushnir VM . GERD phenotypes from pH impedance monitoring predict symptomtic outcomes on prospective evaluation. Neur Gasmotil. (2016) 28:513–21. 10.1111/nmo.12745 PMC4808441 26686239

[40] Guo T Wang Z. Application of portable 24-hour esophageal pH-impedance monitoring in analyzing acid exposure patterns, reflux types, and characteristics in rGERD patients. Lab Med Clinic. (2018) 15:1171–3.

[41] Dai Jing Gao Feng Esophageal Function Tests in Non-Erosive Reflux Disease vs. Reflux Hypersensitivity 2017 Annual Conference of Chinese Journal of Hospital Pharmacy Qingdaom, 2017

[42] Lv B. Are refractory gastroesophageal reflux symptoms always GERD? Chin J Gastroenterol. (2017) 22:129–32.

[43] Boeckxstaens GE Smout A. Systematic review: role of acid, weekly acidic and weekly alkaline reflux in gastro-oesophageal reflux disease. Aliment Pharm Ther. (2010) 32:334–43. doi: 10.1111/j.1365-2036.2010.04358.x 20491749

[44] Yu Y Yu X Yao J Zou J Wang Y Xiang D . Analysis of impedance-pH monitoring results in 36 patients with refractory gastroesophageal reflux disease. Chin J Gastroenterol. (2012) 17:347–50.

[45] Li H Wang A Xu L. Significance of acid reflux, weakly acidic reflux, and weakly alkaline reflux in gastroesophageal reflux disease. J Gastroenterol Hepatol. (2015) 4:485–7.

[46] Wang L Miao Y. Research advances in refractory gastroesophageal reflux disease. Chin J Gastroesoph Reflux Dis. (2016) 3:179–82.

[47] Kahrilas PJ Boeckxstaens G. Failure of reflux in hibitors in clinical trials: bad drugs or wrong patient. Gut. (2012) 61:1501–9. 22684485 10.1136/gutjnl-2011-301898

[48] Khan MQ Alaraj A Alsohaibani F Al-Kahtani K Jbarah S Al-Ashgar H. Diagnosticutility of impedance-pH monitoring in refractory non-erosive reflux disease. J Neurogastroenterol Motil. (2014) 20:497–505. doi: 10.5056/jnm14038, 25273120 PMC4204403

[49] Szallasi A Blumberg PM. Vanilloid (capsaicin) receptors and mechanisms. Pharmacol Rev. (1999) 51:159–212. 10353985

[50] Shieh KR Ch Y Liu T. Evidence for neurotrophic factors associating with TRPV1 gene expressionin the inflamed human esophagus. Neurogastroenterol Motil. (2010) 22:971–e252. 20518854 10.1111/j.1365-2982.2010.01530.x

[51] Cheng L de Monte S Ma J. HCl-activated neural and epithelial vanilloid receptors (TRPV1) in cat esophagealmucosa. Am J Physiol Gastrointest Liver Physiol. (2009) 297:G135–14. doi: 10.1152/ajpgi.90386.2008 19389802 PMC2711757

[52] Fujino K de la Fuente SG Takami Y Takahashi T Mantyh CR. Attenuation of acid induced oesophagitis in VR-1 deficient mice. Gut. (2005) 55:34–40. doi: 10.1136/gut.2005.066795 16091555 PMC1856385

[53] Vakil N van Zanten SV Kahrilas P Dent J Jones R Global Consensus Group. The Montreal definition and classification of gastroesophageal reflux disease: a global evidence-based consensus. Am J Gastroenterol. (2006) 101:1900–20. doi: 10.1111/j.1572-0241.2006.00630.x 16928254

[54] Guarino MP Cheng L Ma J Harnett K Biancani P Altomare A . Increased TRPV1 gene expression in esophageal mucosa of patients with non-erosive and erosive reflux disease. Neurogastroenterol Motil. (2010) 22:746–751, e219. 20456759 10.1111/j.1365-2982.2010.01514.x

[55] Kahrilas PJ Sifrim D. High-resolution manometry and impedance-pH/manometry: valuable tools in clinical and investigational esophagology. Gastroenterology. (2008) 135:756–69. doi: 10.1053/j.gastro.2008.05.048, 18639550 PMC2892006

[56] Bielefeldt K Davis BM. Differential effects of ASIC3 and TRPV1 deletion on gastroesophageal sensation inmice. Am J Physiol Gastrointest Liver Physiol. (2008) 294:G130–8. doi: 10.1152/ajpgi.00388.2007 17975130

[57] Liu HF Zhang JG Li J Chen XG Wang WA. Improvement of clinical parameters in patients with gastroesophageal reflux disease after radiofrequency energy delivery. World J Gastroenterol. (2011) 17:4429–33. doi: 10.3748/wjg.v17.i39.4429, 22110270 PMC3218158

